# Encapsulation of Natural Bioactive Compounds by Electrospinning—Applications in Food Storage and Safety

**DOI:** 10.3390/polym13213771

**Published:** 2021-10-31

**Authors:** Bogdănel Silvestru Munteanu, Cornelia Vasile

**Affiliations:** 1 Faculty of Physics, Alexandru Ioan Cuza University, 11 Carol I Bvd, 700506 Iasi, Romania; muntb@uaic.ro; 2 Laboratory of Physical Chemistry of Polymers, “P. Poni” Institute of Macromolecular Chemistry, Romanian Academy, 41A Grigore Ghica Voda Alley, 700487 Iasi, Romania

**Keywords:** essential oil, bioactive compounds, electrospinning, encapsulation

## Abstract

Packaging is used to protect foods from environmental influences and microbial contamination to maintain the quality and safety of commercial food products, to avoid their spoilage and to extend their shelf life. In this respect, bioactive packaging is developing to additionally provides antibacterial and antioxidant activity with the same goals i.e., extending the shelf life while ensuring safety of the food products. New solutions are designed using natural antimicrobial and antioxidant agents such as essential oils, some polysaccharides, natural inorganic nanoparticles (nanoclays, oxides, metals as silver) incorporated/encapsulated into appropriate carriers in order to be used in food packaging. Electrospinning/electrospraying are receiving attention as encapsulation methods due to their cost-effectiveness, versatility and scalability. The electrospun nanofibers and electro–sprayed nanoparticles can preserve the functionality and protect the encapsulated bioactive compounds (BC). In this review are summarized recent results regarding applications of nanostructured suitable materials containing essential oils for food safety.

## 1. Introduction

Packaging is used to protect foods from environmental factors and microbial contamination to maintain food quality and safety [1]. Food spoilage or poisoning directly affecting public health can be reduced through bioactive packaging, which extends the shelf life of perishable food particularly those susceptible to microbial alteration [2].

Unlike modified atmosphere packaging where the role is only to restrict exchanges of CO_2_, O_2_, water vapor, and aromatic compounds between the food and its external or local environment [3], bioactive packaging provides antibacterial and antioxidant activity with the goal of extending shelf life and the safety of food [4].

The development of active/bioactive materials aiming to the maintaining or enhancing the safety and quality of packaged food by the incorporation of antimicrobial natural compounds and/or antioxidant natural compounds [5] is now an active research area [6,7,8,9,10]. Unfortunately, their use in natural form in food packaging materials in foods is restricted because of their low stability against temperature, oxygen, or light exposure during processing of the food, distribution, and storage [11]. Also, their uncontrolled release profiles can significantly deteriorate their biological benefits [11]. To overcome these limitations, appropriate carriers and encapsulation techniques were designed.

The natural bioactive compounds (BCs) with antimicrobial and antioxidant activities as essential oils, some polysaccharides [12,13], natural inorganic particles (oxides, nanoclays, metals, such as silver) [14] into food packaging can protect food from microbial alteration and extend shelf life, reducing economic losses and health issues caused by foodborne pathogens [15,16].

## 2. Essential Oils

Essential oils (EO) are natural substances extracted from plants herbs and spices such as garlic, black cumin, cloves, cinnamon, thyme, basil, bay leaves, coriander, mustard, rosemary, sage and others [17] as complex mixtures of aromatic and volatile organic compounds [18]. They resulted as secondary metabolites that plants produce for protection by acting as insecticidal and antimicrobial agents or for attracting insects for flower pollination [18].

The essential oils are obtained from different parts of the aromatic plants (mainly the flowers and leaves) but almost every part of a plant such as stems, roots, flowers, leaves, fruits, stem bark and even in seeds. EOs can be extracted in different ways [19].

The essential oils are highly volatile, lipid-soluble liquids [20]. They are also soluble in organic solvents such as ethanol, acetone, and methanol [21]. Their density is less than 1, with few exceptions (cinnamon, sassafras, clove, vetiver EOs) [22].

Due to the constituents of the essential oils, they have a wide range of antimicrobial, antioxidant activities [23]. Furthermore, many of these volatile constituents have antifungal effect against yeasts as well as filamentous fungi, being potentially useful as food preservatives [24,25].

By their antioxidant activity they are also beneficial in various food industries for preserving food against the lipid peroxidation caused by the free radicals on fats and oils [26]. Lipid peroxidation usually results in unpleasant odors and flavors of foods causing deterioration of the food quality and also decreasing the nutritional value of food [27]. Due to their perceived safety profiles EOs as natural antioxidants are favored over the synthetic antioxidants such as butylated hydroxyanisole and butylhydroxytoluene whose applicability have been discouraged due to safety, health, and environmental concerns [28].

Complex mixture of components of EOs that give a characteristic odor and flavor to the plants are usually rich in phenols, esters, terpenes, sesquiterpenes, aldehydes, ethers, peroxides, alcoholic compounds, phenylpropanoids, among others [22,25].

Constituents of the essential oil such as thymol, carvacrol, γ-terpinene, eugenol have antioxidant effects [29] while other constituents such as limonene, eugenol, pinene, carvone, and linalool carvacrol have been suggested as agents responsible for the antimicrobial efficiency against food-borne pathogens [30]. Eugenol exhibited rapid bactericidal activity against *Salmonella enterica* serovar *Typhimurium*, terpineol had good bactericidal effect against *S. aureus* strains, carveol, citronellol and geraniol presented a rapid bactericidal activityagainst *E. coli* [31]. The higher antimicrobial activity was explained by the presence of hydroxyl groups (alcohol and phenolic compounds) [31]. The compounds such as benzoic acids, benzaldehydes, and cinnamic acid have shown up to 50% inhibition of *Listeria monocytogenes* under anaerobic conditions [32].

The EOs of similar plants have been reported to have differences in composition depending on the geographical location that the plant is found [33]. Also, EOs composition and yield can vary with habit conditions and climate, harvesting stages, planting, and preparation methods, plant age and genetics [34]. Weather parameters such as rain and the temperature of the atmosphere have been found to influence the content of the oil and the composition of the aromatic plants [35].

The main components of some EOs recently studied are presented in Table 1.

## 3. Encapsulation

EOs and other active compounds, in natural form, have restricted applicability [55] because of their poor stability, as they are easily degraded [56] by oxidation, hydrolysis, crystallization or enzymatic deterioration, during storage or processing in harsh conditions in the presence of oxygen and light [57]. Also, it is important to consider that these active compounds have low thermal stability, since high temperature used during food processing causes loss of their functionalities [58]. This can significantly deteriorate their flavor, solubility and biological benefits as is the case with the pomegranate peel extract which is currently affected by color and instability issues associated with easy oxidation [59] or the EO of *Satureja hortensis* which drastically changes its composition through the heating of the samples over 160 °C [60]. Also, volatility [55] and low water solubility [61] are associated with the EOs when exposed to air, which limits its application in food preservation [62]. The strong and intense flavor of EOs may be transferred as taste to the packed food [63,64] as well.

For these reasons, a protection technique is required before the addition of natural EOs (or other BCs) into food systems [65]. Consequently, many researchers have encapsulated them into other protection materials in order to make full use of their anti–oxidant and antimicrobial properties [66].

The research developments in the area of the nanoencapsulation of BCs in food pack–aging materials are continuously growing [67] as the nanoencapsulation [68] can protect the BCs against oxidative degradation upon exposure to air or high temperatures during food processing [69], and can enhance the bioavailability of the BC, releasing them in a controlled manner and preserving their activity [70]. For example, encapsulating thyme EO into β-cyclodextrin/ε−polylysine can reduce undesirable deficiencies such as volatility and hydrophobicity of the BCs [71]. The antimicrobial carvacrol can be protected/encapsulated in a starch fiber matrix, to avoid direct contact with food and reduce the effects on sensorial features [72]. Encapsulation in zein microparticles improved thermal stability of polyphenols from maqui fruit extract when exposed to high temperatures related to processed foods [73]. Orange and thyme oil adsorbed in halloysite or montmorillonite clay and then encapsulated in a polyethylene/polyamide/polyethylene multilayer film prolongated aroma release [74]. Encapsulation of black pepper (*Piper nigrum L.*) EO into sodium alginate and gelatin by complex coacervation avoid the loss of the main volatile from EOs which were preserved (80% of their original content) [75].

There are several methods to encapsulate/protect these sensitive natural bioactive antioxidants/antibacterials (including phenolic compounds, etc.) in food packaging for active compound delivery: films [76,77,78], microencapsulation via the spray-drying (wall materials that suitably protect the inner EOs from oxidation and evaporation) [79], nanoprecipitation [80], but recently, electrohydrodynamic processes [81] “electrospinning” [82] and “electrospraying” [83,84] have received increased attention due to their versatility, cost-effectiveness, and scalable technologies [85,86,87,88].

## 4. Encapsulation by Electrospinning

**Figure 1:**
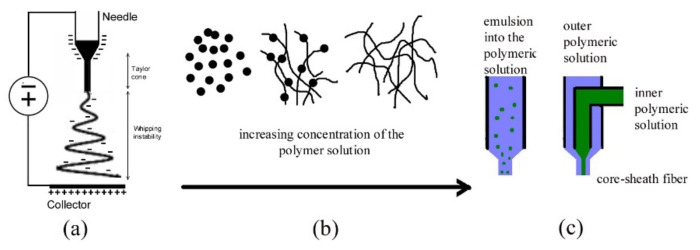
(**a**) Taylor cone and whipping instability in a typical electrospinning setup; (**b**) The same polymer can generate fibers (electrospinning) or droplets (electrospraying) depending on the viscosity of the solution; (**c**) emulsion versus coaxial electrospinning.

A typical electrospinning set-up has a high voltage power supply connected to a metallic nozzle and a metallic collector. When a high voltage is applied between the solution of the polymer and a metallic collector, a drop of a polymer solution ejected at the tip of the nozzle will turn into a conical droplet known as the Taylor cone (Figure 1a), the electrostatic repulsive force acting on the drop surface counteracts the surface tension and a liquid jet is ejected that is deposited onto the collector in the form the nanofiber mesh [89]. Electrospinning and electrospraying can operate at ambient conditions (atmospheric pressure and room temperature) producing micro/nanostructures in dried form in a one-step process [90]. The viscosity of the solution is the dominant parameter which decides if fibers (electrospinning) or droplets (electrospraying) will be obtained (Figure 1b). A too low viscosity results in droplets of polymer (electrospray) due to the interruption of polymeric filaments. The boundary concentration between electrospray and electrospinning depends on the molecular weight of the polymer and the nature of the solvent [91].

The needle electrospinning is inexpensive and versatile but its use in applications is restricted because its low production rate. Also, the needle blocking frequently occurs, particularly with high viscosity polymer and functional nanoparticles/bioactive substances in the spinning solution, which makes it difficult to produce nanofibers continually. Therefore, the needleless electrospinning was developed [92] for mass production of nanofibers. During needleless electrospinning the polymeric multi-jet initiation is a self-initiated process taking place on a free liquid surface and usually rotating disks/cylinders are used to feed the initiated Taylor cones with polymeric solution to keep the electrospinning process continuous and not interrupted [93,94].

A profiled multi-pin electrospinning setup may overcome the limitations of the needleless and needle electrospinning (for example uncontrolled/uneven Taylor cone formation, needle clogging, and the requirement of very high voltage). A profiled multi-pin surface is designed to support the nano/microparticles in the polymer solutions [93]. This increases the range of multifunctional electrospun nanofiber applications by the development of a single matrix with multifunctional characteristics and improved mechanical and electrochemical performances [93].

The most common approaches used to encapsulate bioactive antioxidants/antibacterials into nanofibers are emulsion and coaxial electrospinning (Figure 1c). In both cases the nanofibers generated have an outer polymeric sheath (base polymer) and inner bioactive core, although the processes involved are different: coaxial electrospinning generates core–sheath fibers by physical separation of two polymeric solutions flowing through concentrically aligned nozzles: an outer sheath polymeric solution and the second inner polymeric solution containing the bioactive substance [95]. The emulsion electrospinning involves a single polymeric solution containing an emulsion of the bioactive substances; the subsequent separation of the emulsified droplets into the sheath polymeric phase takes place as the solvent evaporates from the electrospun fibers [96].

The obtained electrospun nanofibers and electrosprayed nanoparticles can serve as protection for the bioactive compounds against any severe conditions (such as high temperatures and/or pressures involved during packaging or food processing, storage, light, oxidation) preserving the functionality of the active compound encapsulated within the electrospun nanofibers [97] as well as controlled delivery/release of bioactive compounds [98]. Their efficiency in maintaining the stability of the bioactive compound [99] enhances the bioavailability and bioactivity during processing, storage and consumption, the encapsulation process alleviating the unpleasant flavor or taste of phenolics [100,101].

For food packaging, the electrospun/electrosprayed nanofibers/nanoparticles can be more efficient than films in view of several advantages such as larger surface to volume ratio, higher crack resistance, interconnective structure, good adhesion properties (in case of coatings) and higher porosity, high loading capacity of the active compounds [102,103,104]. Using this approach, new packaging can be formulated in a single step with the additional advantage of simultaneously and intrinsically producing interlayers [105,106,107] (coatings [108]) with encapsulation performance [109]. This offers several benefits compared to the traditional encapsulation techniques which may be detrimental for the active properties of many of the antimicrobials and antioxidants (EO) due to the high temperatures used for drying the obtained materials [110,111].

Compared to the traditional encapsulation techniques which may be detrimental for the active properties of many of the antimicrobials and antioxidants (i.e., EO) due to the high processing temperatures used for drying the obtained materials [110,111] electrospinning offers the advantage of the absence of heat [100,112,113] during the drying of the structures. As the solvent is evaporated during the flight of the solution towards the collector due to the high voltage application [114], no high temperature applied [113]. This is important for preserving the structure and achieving high encapsulation efficacy of the thermo-sensitive [115] and volatile [113] bioactive substances upon processing and storage [73].

Besides the advantage of low processing/production temperature, the electrospun nanofibers can also show an increase of the thermal stability [116,117,118] during the subsequent thermal processing of the bioactive compounds, which are known to be highly sensitive to thermal treatments [119] (polyphenols, principally anthocyanins) [73]. For example, volatile bioactive substances (carvacrol [72]) encapsulated in the nanofibers have greater thermal stability than in the free form, which broadens the processing temperature range. Similar results were found in carvacrol and thymol loaded zein nanoparticles [120]: an enhanced thermal stability of EOs loaded-zein nanofibers was a consequence of the interaction [121] between the polymer and EOs, which leads to a higher heat resistance of the resulting nanofibers, compared with the unprotected EOs. Electrosprayed hydroxypropyl-β-cyclodextrin microcapsules containing maqui fruit extract were successfully obtained and had lower polyhenolic content reductions when exposed at high thermal conditions simulating baking conditions compared with the non-encapsulated samples [73]. Chitosan/polycaprolactone electrospun nanofibers with chlorogenic acid loaded halloysite nanotubes (HNTs) had improved thermal stability due to the hydroxyl groups present in the cavity of HNTs interacting with bioactive molecules via hydrogen-bonds for efficient encapsulation and controlled release [122,123]. Immobilized enzymes present higher thermal stability than the free enzymes [124,125], which makes the immobilization in crosslinked fibers to be effective in increasing thermal stability of enzymes which is beneficial for applications in which food products are subjected to high temperatures [72,124].

## 5. Applications of Nanofibers Containing Essential Oils for Food Safety

As natural active compounds in food industry, EOs and plant extracts have attracted considerable research, because they have demonstrated biological activities of which the most important are the antimicrobial, antioxidant activity, [126,127]. For example, EOs from bay (*Laurus nobilis*) and rosemary (*Rosmarinus officinalis*) have been studied as natural food preservatives because of their antimicrobial and antioxidant activity [128,129]. Biodegradable active food packaging structures with good water and thermal stability based on hybrid cross-linked electrospun polyvinyl alcohol electrospun nanofibers containing essential oils from *Laurus nobilis* and *Rosmarinus officinalis* were successfully tested to chicken fillets [130]. Pomegranate peel extract is rich in polyphenols, including a wide variety of tannins, exhibits antibacterial activity because the morphology of microorganisms is modified by precipitating proteins, causing cell membrane leakage and cell lysis [131,132]. Chitosan films incorporated with *Plectranthus amboinicus* EO had antimicrobial activity against food pathogens, together with good water vapor barrier [133]. Antioxidant films with reduced water vapor transmission rate obtained by coating the polylactic acid (PLA) substrate with chitosan enriched with 1% and 2% rosemary EO delayed the lipid oxidation of raw chicken meat [134]. Chicken breast samples wrapped with starch films incorporated with 1.0% rosehip extract had lower peroxide values compared with non- rosehip extract films as well as the non–packaged control, suggesting that lipid oxidation in the chicken breast is inhibited by the inclusion of rosehip extract [135]. Addition of 1.5% cymbopogon citratus EO to chitosan film (solvent casting) increased water vapor permeability about 30%, decreased film solubility in water and extended meat shelf life as total bacterial count was in acceptable range after 10 days of storage [136].

Besides the EO, among the active compounds that have received attention recently are spices, herbs, chitosan and its mixtures [137], bacteriocins etc. [138].

Various antimicrobial/antioxidant electrospun nanofibers containing EOs or other BCs with applications in food preservation are presented in Table 2.

The higher surface to volume ratio and porous structures generated through electrospinning [146], have a beneficial effect for the long-term application of antimicrobials in comparison with the film casting approach [113]: For example, after 28 days of storage (at the end of the storage time), the zein nanofibers loaded EOs showed significantly lower bacterial counts than the zein cast films containing the same amount of EO (from *Laurus nobilis* and *Rosmarinus officinalis*). The morphology of the obtained structures had a significant effect regarding the long–term release (sustained antimicrobial activity), indicating the efficiency of the encapsulation to protect the active compounds by slowing down their volatilization [113]. The fast release of EO from the zein cast film expeditiously reduced the bacteria at short storage times. However, later on the effect was reduced [113].

Enhanced interactions/compatibility between the bioactive compounds and the encapsulating materials can sustain the release of antimicrobial agents over longer time from the fiber mats. For example electrospun nonwovens containing 30% carvacrol encapsulated in starch sustained antimicrobial activity for at least 30 days against *L. monocytogenes*, *Salmonella Typhimurium*, *E. coli* and *S. aureus*. [72] due to the interactions between starch and carvacrol (evidenced by FT-IR and the increased viscosity due to the carvacrol addition). Therefore, the starch nanofibers are auspicious materials to be used as a vehicle for carvacrol release in antimicrobial and antioxidant food packaging [72].

The addition of green tea extract to the protein (gelatin or zein) nanofibers encapsulating curcumin resulted in strong interactions with the proteins (gelatin), which improved the protective effect of the fibers and slowed down the curcumin release in hydrophobic food simulants (although it did not prevent their collapse in water) [147]. Due to the poor solubility of curcumin in aqueous media, it was developed a strategy based on its incorporation through liposomes, which allowed the successful incorporation of the curcumin into gelatin fibers. Very high encapsulation efficiencies were attained for both zein and gelatin, with zein showing an augmented preservation effect [147].

The low and sustained release of antimicrobial agents from the nanofiber mats is expedient since it minimizes bacterial colonization for a longer period of time [148,149]. Thus, for chitosan/poly(ε-caprolactone) nanofibers containing oregano essential oil most of the oil (55–80%) was not released after 96 h, which demonstrates the durability of EO in the electrospun fiber mats [148]. In the kinetic release profiles, before the long and steady plateau indicative of EO release by diffusion from the bulk of the fibers, an initial burst release related to the near-to-surface residing oregano essential oil molecules can be observed [148]. In addition, as the content of the oregano essential oil increased, the amount of released oil from the fibrous mats increased [148,150].

## 6. Base Polymers Used to Encapsulate Active Substances in Nanofibers

The traditional packaging mainly consisting of plastic materials is a considerable interest because the packaging wastes are non–degradable [151]. Biodegradable bio-based materials with biodegradability and nontoxicity have attracted attention as a sustainable alternative for the development of biodegradable and active food packaging due to the environmental benefits [152]. The most used biodegradable biopolymers for electrospinning are extracted/derived from biopolymers/biomass starches, cellulose, cellulose acetate, chitin, chitosan, proteins (gelatin, zein, silk) [102,153], bio–engineered polymers (such as poly(hydroxy alkanoates (PHAs), [poly(glutamicacid) which are bio-synthesized using microorganisms and plants], [154,155], obtained from bio-derived monomers such as polylactic acid [154,155]. Synthetic biopolymers such as poly(ethylene glycol) (PEG), poly (vinyl alcohol) (PVA), poly(ethylene oxide) (PEO), poly(caprolactone) (PCL), are also used [156].

Among the proteins, the zein has attracted attention as a result of its good properties such as toughness, flexibility, compressibility, hydrophobicity, nontoxicity, and low cost [157,158]. Also, gelatin, as a natural biomolecular polymer extracted from connective tissue in animals, is chosen due to its high biosecurity [159]. As a synthetic biopolymer, polyvinyl-alcohol is a water soluble polymer with biodegradability, non-toxicity, biocompatibility, very good optical properties, and good film-forming ability which make it useful for the development of active food packages or coatings [160,161].

Among the natural polymers chitosan is nontoxic, edible, and biodegradable derived by deacetylation of chitin which is the second most abundant biopolymer in nature after cellulose. Besides its antibacterial and antioxidant activity, chitosan has several advantages including its exceptional biocompatibility and biodegradability [162], good film-forming properties, nontoxicity, which make chitosan and chitosan-based carriers [163] suitable for use as active coatings or film material in different food packaging [164,165] or as a functional/active ingredient to improve the shelf lives of food products [166]. It can be used as an antioxidant [167] and antibacterial [168] agent and polymer substrate simultaneously [76]. From an environmental point of view, chitosan nanofibers as packaging materials are made from sustainable sources, bio–friendly, and inherently biodegradable. Incorporating/encapsulating EO as antimicrobial and antioxidant natural extracts to chitosan films and coatings, can improve the functionality of packaging (shelf−life extension of food products by controlling the active components release into the surface) [169]. For example, chitosan films incorporated with essential oil from *Plectranthus amboinicus* had antimicrobial activity against food pathogens [133].

Antibacterial packaging nanofibers based on chitosan [170] can preserve the quality and safety of the food products during distribution and storage due to their physicochemical properties [171]. Blend of electrospun chitosan (CS)/poly(ε-caprolactone) (PCL) containing 5% oregano had antibacterial activity against Gram-positive (*Staphylococcus aureus, Listeria monocytogenes*) and Gram−negative (*Salmonella enteritidis, Escherichia coli*) bacteria [172]. PLA/Chitosan fibres containing cinnamon EO had a high antibacterial activity against *Escherichia coli* and *Staphylococcus aureus* due to the long-term cinnamon EOs release [173]. In another study thin chitosan cast films laminated with electrospun nanofibers containing *Zataria multiflora* and cinnamon EO produced for active food packaging applications with antifungal antioxidant properties [174].

## 7. Encapsulation Efficiency in Electrospun Nanofibers

Encapsulation efficiency (EE) of EOs in electrospun fibers depends on a number of factors, like electrospinning configuration (uniaxial vs. coaxial), the type and the molecular weight of the electrospun polymer [175], size distribution of the fibers [176].

The EE is also affected by the physical interactions between core and encapsulating materials [177] which are governed by the chemical nature of the electrospun and the encapsulated materials (active substance and fiber material) [178,179]. For example, the presence of apolar amino acids in zein enables interactions with oil constituents, which makes zein to be an appropriate matrix for EO encapsulation due to its amphiphilic nature and exhibiting high EE [180].

The decrease in EE upon increasing bioactive concentration it is well known and is caused by the less efficient coating of the bioactive compound (BC) as the ratio BC/matrix increases or due to limitations in the loading capacity of the obtained structures [181]. It is possible to develop electrospun fibers incorporating up to approx. 30% oil [112], although quantities above the 10% may produce inadequate fiber morphologies [182] as the increased content of the encapsulated material decreases the EE. The volatility of the encapsulated bioactive substances also influences the EE for the reason that lower EE value can be associated with higher volatility of EO [183,184].

In many situations, as in controlled release food packaging using BC (or EO) loaded into nanofibers, the dispersion of the EO in a polymer solution (emulsion electrospinning) is considered sufficient [185] so a number of publications have focused on the direct blending of BC with spinning solutions to obtain electrospun nanofibers. However, the release of the BC from the nanofibers is problematic due to the initial burst release of the encapsulated bioactive compounds physically absorbed on fiber surface [186]. For example, curcumin encapsulated into electrospun gelatin nanofibers exhibited a burst release from the fiber surface (70% release after only 4 h) [153]. Caffeine incorporated in cellulose spinning solution had a fast release of 60% caffeine from the nanofibers in aqueous solution [187].

**Figure 2:**
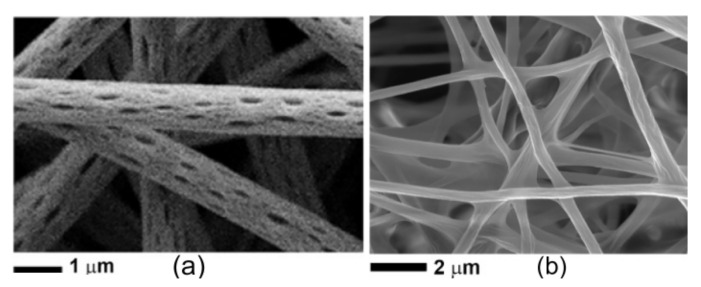
SEM micrographs of PLA-BP fibres uncoated ((**a**) magnification scale 1 μm) and ((**b**) magnification scale 2 μm) coated with chitosan [189]. Reprinted with permission from ref. [189]. 2021 MDPI, Basel, Switzerland, 2021.

Also, the surface of electrospun nanofibers may be influenced by the presence of the emulsion of the BC due to the phase separation during the electrospinning process, as a consequence of the evaporation of the most volatile components of EOs and their poor miscibility with the polymer [188]. The addition of black pepper EO to the PLA/acetone solution resulted in fibers with surface nano-pores elongated along the fibers, which is the direction of polymer stretching during the electrospinning process—Figure 2. Despite the evaporation of some chemical constituents of EOs during electros pinning, the oil retained its antibacterial activity suggesting that the volatile components of the EO were evaporated only at the fiber surface. In order to preserve the oil functionality, the fibers were coated with a thin layer of medium molecular weight chitosan by immersion in the chitosan solution. In this way the presence of the chitosan coating limited the extraction in ethanol of the black pepper EO [189].

The core-shell nanofibers fabricated by coaxial electrospinning offer one solution to avoid the initial burst release of the bioactive compounds physically absorbed on fiber surface when emulsion electrospinnig or direct blending of bioactive compounds with spinning solutions is used [185]. Another solution to avoid the burst effect and to provide a sustained release is the encapsulation within a nano-carrier (e.g., nanotubes, nanoparticles, nanoemulsions) and subsequent loading (emulsion electrospinning) of the EO/nano-carrier into electrospun nanofibers [190,191].

For example, cyclodextrins can be used to encapsulate food additives and essential oils (guest molecules) by non-covalent host-guest inclusion complexes due to their hydrophobic (host) cavity. Cyclodextrins are non−toxic and biodegradable cyclic oligosaccharides produced by enzymatic degradation of starch which are composed of α-1,4-linked glucopyranose units forming a truncated cone-like structure. Thymol/gamma-cyclodextrin inclusion complex together with zein were electrospun in uniform and bead-free zein nanofibers, which were effective at reducing the bacterial count in meat stored up to 5 days at 4°C. [103]. In the same respect, thyme EO/β-cyclodextrin/ε-polylysine nanoparticles exhibited better and prolonged antimicrobial activity than the free thyme EO due to the encapsulation of thyme EO into β-cyclodextrin cavity and presence of ε-polylysine [71]. Also, an efficient antioxidant nanofibrous material was obtained by loading quercetin/gamma-cyclodextrin inclusion complex into electrospun zein nanofibers [192]. Liposome-encapsulated Tea tree oil loaded into chitosan nanofibers had antibacterial effect against *Salmonella enteritidis* and *Salmonella typhimurium* whitout corrupting the sensory properties of chicken meat [193]. Due to inclusion complexation the pullulan nanofibers containing eugenol encapsulated in γ-cyclodextrin preserved ~93% of the volatile essential oil (compared with ~23% in case of pullulan/eugenol nanofibers). The inclusion complexation also increased the thermal stability for eugenol and preserved the antioxidant activity of the nanofibers even after 3 months’ storage at room temperature (~98%) and heat-treatment at 175 °C for 1 h (~93%) [81].

**Figure 3:**
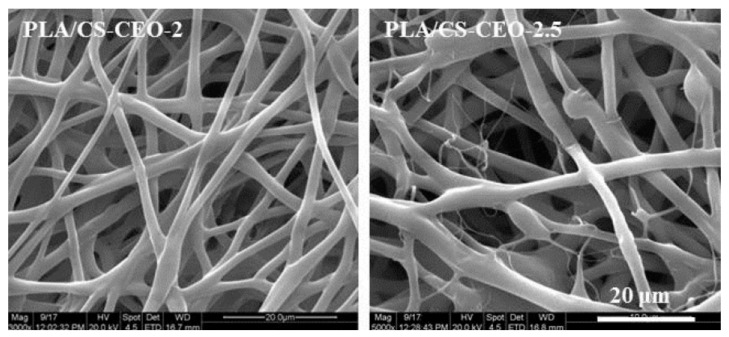
Poly (lactic acid) fibers containing beads of cinnamon essential oil encapsulated into chitosan nanoparticles (magnification scale 20 μm) [194]. Reprinted with permission from ref. [194] 2017 MDPI, Basel, Switzerland, 2017.

Essential oils were also encapsulated into chitosan nanoparticles which were subsequently loaded into polymeric fibers. For example, emulsion electrospinning was used to obtain poly(lactic acid) (PLA) nanofibers loaded with cinnamon EO previously encapsulated into chitosan nanoparticles. The obtained nanofibers showed high long-term antibacterial activity against *Escherichia coli* and *Staphylococcus aureus* due to the sustained release of cinnamon EO. When the concentration of chitosan- cinnamon EO nanoparticles in the PLA solution was increased to 2.5%, the obtained PLA fibers had a smaller diameter and was observed theformation of beads along the fibers (Figure 3) [194].

## 8. Crosslinking of the Electrospun Nanofibers

Despite all the mentioned advantages, some of the drawbacks of certain matrix polymers used for electrospinning (chitosan, polyvinyl alcohol, zein) are their weak mechanical properties and more importantly their high−water solubility and rapid dissolution rate which affects the fiber intergrity and limits the applications in real foods with high water activities [195,196]. In this respect, the crosslinking is a process used to perserve some characteristics of the base material, such as decreased water solubility and improved mechanical properties [197,198].

Also, crosslinking delays the release of active compounds caused by the ultrathin structure of the nanofibers. However, after the crosslinking process, the fibers’ porosity is reduced [199], limiting the accessibility to the bioactive substance [124,157] which may even cause its inactivation [124]: For example, the enzyme (α-amylase) linking to the polyvinyl alcohol (PVA) molecule promoted by the crosslinking (using vapor phase glutaraldehyde), decreased its enzymatic activity down to 50% after 2 hr. of crosslinking, and after 14 days of storage, decreasing with the crosslinking time. The authors observed that after crosslinking, nanofibers became densely packed with a compact inter-fiber network, due to the adherence between the PVA fibers, and as a result, the surface area was reduced, making the substrate access to the enzyme more difficult [124]. Moreover, the fibers still maintained part of their fibrous morphology even though they appear practically fused, suggesting that the crosslinking took place throughout the fibrous conformation [124].

Another benefit of the crosslinking is the reduced interstitial spaces between the biopolymer chains, which reduces the molecular motion and prevents extensive swelling [200] of the electrospun fibers [201]. For example, the crosslinked nanofibers based on *Colocasia esculenta tuber* protein/chitosan/poly(ethylene oxide) maintained their structure after immersion in phosphate buffered saline. The crosslinked nanofibers resulted in higher ultimate tensile strength and lower ultimate strain compared to the non-crosslinked nanofibers [202]. Also, the crosslinking with various crosslinking agents improved the functionality and applicability of water soluble based matrices [203,204] such as the PVA/EO uncrosslinked nanofibers whose fibrillar morphology is completely lost after water immersion [205,206,207].

Crosslinking can increase the thermal stability of the encapsulated active substances. For example, in order to overcome its low thermal stabilty, cabreuva EO (extracted from the wood of *Myrocarpus fastigiatus*) was encapsulated into nanoparticles of crosslinked chitosan (with sodium citrate) before loading it into PVA nanofibers. The electrospinning process maintained the structural configuration in which the essential oil was initially confined as the thermogravimetric analysis did not detect any loss mass corresponding to essential oil evaporation [208].

Crosslinking can be achieved either by physical procedures such as heat treatment, gamma or ultraviolet irradiation, or by chemical agents (glutaraldehyde, formaldehyde) [209]. However, most of these crosslinking chemical agents have been reported to be harmful. As an example, glutaraldehyde is highly toxic and are likely to escape from the packaging material into the food, with negative impact on food safety [210,211,212].

Therefore green, non-toxic crosslinking agents are proposed such as genipin found in gardenia fruit extract and which may become a preferable alternative to glutaraldheyde [213] or polybasic organic acids [214]. Also, citric acid as a food-grade antioxidant [215], [216], was successfully incorporated as a natural cross-linker for PVA to promote the crosslinking of the electrospun PVA matrix, in order to avoid the disintegration upon water immersion [217]. These crosslinked active food packaging structures containing EO and citric acid had improved water resistance and thermal stability [217] with respect to their non-crosslinked counterparts [130]. The crosslinked films maintained their fibrillar morphology in water even if a certain swelling was observed) [130]. The PVA/EO samples inhibited the lipid oxidation (up to 70%) and displayed antimicrobial activity when applied onto chicken breast fillets, having a positive effect on both the pH and color parameters during storage [130]. It was also shown that the addition of adipic acid as a crosslinking agent could improve the compatibility and interface reaction between polysaccharide from *Dendrobium officinale* and polyethylene oxide due to the grafting reaction between carboxyl groups in adipic acid and hydroxy groups in polysaccharide, which was beneficial for the reduction of the differences in polarity between polymers [218,219]. Addition of adipic acid increased the viscosity which resulted in uniform and smoother fibers at high polysaccharide content together with antibacterial activity against *E. coli* on pork samples, without impact on the quality of pork meat [218].

Polyphenols were reported to act as natural cross-linkers for proteins [220], since they can strongly interact with them through hydrogen bonding and hydrophobic interactions [221]. For example, when green tea extract was incorporated within the gelatin fibers containing curcumin, an improved curcumin protection was observed together with a delay in the release of curcumin from the gelatin coatings which was attributed to the intermolecular interactions established between the polyphenol rich-extract and the protein [147]. There are also recent studies which reveal that numerous food wastes, particularly fruit and vegetables by-products, are a good source of bioactive compounds that can be extracted and reintroduced into the food chain as natural food additives or in food matrices for obtaining nutraceuticals and functional foods [222].

## 9. Polysaccharides as Antimicrobial Polymers for Electrospinning

Polysaccharides are macromolecules composed of sugar units linked by glyosidic bonds which can be found directly in some plants (starch, cellulose, pectin, hemicellulose, gums), algae (agar, alginates), animals (chitin, chitosan, hyaluronic acid) or can have bacterial (dextran, xanthan, cellulose) or fungal (pullulan, yeast glucans) origin [223]. Polysaccharides were explored for their antimicrobial properties: their antibacterial mode of action was shown to be via damaging cellular structural and inhibiting bioenergetics metabolism [224]. A variety of polysaccharides, such as chitosan, dextran, hyaluronic acid, cellulose, other plant/animal-derived polysaccharides and their derivatives have been studied for antimicrobial applications [225] and also, as matrices for incorporate BC into nanofibres, as it was shown above. Polysaccharides are naturally widespread, safe, bio–compatible, residue free.

Amongst natural polysaccharides, chitosan was extensively used as base polymer for electrospinning/electrospraying. Nanofibers based on chitosan (pure or blended with other materials to improve its processability) can be used to obtain various antimicrobial and biodegradable composites, membranes, films which may be subsequently crosslinked in order to increase their strength [226]. These nanofibers can be functionalized with bioactive agents or nanoparticles and used as films exhibiting excellent antioxidant and antimicrobial properties for a variety of food products [227].

For example, antibacterial nanofiber films were prepared by electrospinning gelatin, chitosan, and 3-phenyllactic acid which can be used as an active food packaging [228]. Chitosan/poly (ethylene oxide) (PEO)/lauric arginate composite electrospun nanofibrous films showed increased antimicrobial activity against *Escherichia coli* and *Staphylococcus aureus* depending on lauric arginate concentration. The formation of electrostatic and hydrogen bonding interactions induced by the lauric arginate addition changed the inter- and intramolecular interactions between PEO and chitosan which influenced the mobility of the polymer molecules, increased the crystallinity and decreased melting point [229].

Stable silver nanoparticles produced by chitosan mediated green synthesis were blended with polyvinyl alcohol to form electrospun fibrous composite nano-layers which showed bioactivity, extended the meat shelf-life by inhibiting microbial degradation of packaged food due to the cooperative antibacterial activities of chitosan the silver nanoparticles [230].

Chitosan blended with polylactic acid (PLA) was electrospun as fiber layer on to the surface of a low-density polyethylene (LDPE)/PLA film to produce bilayer antibacterial films. The addition of the chitosan on the bilayer film resulted in higher antibacterial activities with reduced oxygen and water vapor permeability of the LDPE/PLA substrate [231].

Applications of cyclodextrins to obtain nanofibres for food applications was also above presented.

## 10. Inorganic Bioactive Compounds

*Montmorillonite* composite/nylon 6 electrospun nanofibres were deposited over polypropylene films [232] to increase their barrier properties against oxygen and moisture, to reduce moisture absorption and lipid peroxidation in packaged food (potato chips). Shelf life of bread was extended by 2 days using such composites as packaging materials. The malondialdehyde levels in potato chips increased from 0.15 to 0.95 μM g^−1^ suggesting lower rancidity of chips due to less oxygen permeability. Coating of polypropylene films by electrospinning technique has many advantages for food packaging industry because a very low amount of raw material is required to make uniform coating on substrate, composite fibres can by applied for various packaging applications, with coating thickness in nano range on the surface of conventional packaging films [232].

The inclusion of inorganic nanoparticles such as nanosilica and nanoclay into electro spun biopolymeric matrices as polymeric nanofibers enhances and improves the mechanical properties physical and biological properties of polylactic acid [233]. They can act as nucleating agents being considered as special fillers for polymers. It was mentioned above that the usage of biopolymers in developing biodegradable food packaging films as sustainable and safe towards environment is restricted because of the poor mechanical and barrier properties of the biopolymers. By incorporation into PLA of different types (montmorillonite and halloysite) in optima concentration of 3 wt.% nanoclays resulted in the improved mechanical and oxygen barrier properties due to the strong interaction between nanoclays and tortuous path length created by nanoclays respectively [234].

The metal extensively used in food preservation is *silver*. Silver nanoparticles (Ag NP) based-antimicrobial agents show very wide applications, including biomedical applications, as surface treatment and coatings, in chemical and food industries, and for agricultural productivity. Their antimicrobial activity depends on size, shape, and chemical composition, which affect the surface interaction/state of Ag NP [235,236].

AgNPs synthesized from black grapes peel extract have been used to prepare AgPVA nanofibers with good antibacterial activity against *Bacillus cereus*, *Staphylococcus aureus*, *Escherichia coli*, and *Pseudomonas aeruginosa* that increased the shelf life and prevented the decaying caused due to food pathogens when surface-coated over lemon and strawberry [237].

Electrospun nanofibres of cellulose acetate, poly(vinyl chloride), cellulose acetate (CA) or blends of chitosan/poly-(ethylene oxide) containing AgNP [238,239,240] had antimicrobial or antifungal activity against *Staphylococcus aureus* ATCC 29,213, *Propionibacterium acnes* ATCC 6919, and Gram-negative bacteria, such as *Escherichia coli* ATCC 25,992 and *Pseudomonas aeruginosa* ATCC 17,933 which makes them potential materials for the development of active packaging that could extend the shelf life of perishable foods. Nanocomposite nanofibres of PLA/Ag-NP/VitaminE nanofibers inhibited growth of *Escherichia coli*, *Listeria monocytogenes* and *Salmonella typhymurium* up to 100% [241].

Most in vitro studies demonstrated the size-, dose- and coating-dependent cellular uptake of AgNPs, their biodistribution and both in vitro and in vivo toxicity following various routes of exposure showed Ag accumulation and toxicity to different organs. Electrospun nanofibers are capable of improving several attributes of chemical (bio)sensors used to monitor quality of food products and for and agricultural applications, due to the high specific surface area, high porosity and 1-D confinement characteristics.

Electrospun antimicrobial fibrous membranes based on PLA biopolymer containing ZnO provided UV light barrier an antibacterial effect against *Escherichia coli* and *Staphylococcus aureus*. The ZnO addition (with an optimum content of ZnO 0.5 wt.%) improved the mechanical properties of the nanocomposites making them suitable for food packaging applications [242]. A bilayer film with antioxidant and antimicrobial activity was developed from biodegradable polymers with an outer extruded layer of thermoplastic starch and ZnO nanorods and the inner layer of poly(vinyl alcohol) electrospun fibers containing rosemary polyphenols. ZnO nanorods in the outer layer inhibited the growth of *Escherichia coli* over its surface, while rosemary polyphenols included in the inner mat showed an antioxidant activity in food simulant. Furthermore, the inner layer hindered Zn(II) migration from the outer layer towards food simulant and decreased the water vapor permeability by 42% compared to the pure thermoplastic starch film [243].

## 11. Films or Coatings Incorporating Essential Oils: Applications for Food Safety

Besides the encapsulation in nanofibers, the EOs can be incorporated in films and sheets or as coatings to improve the quality of food or polymeric materials destined to food preservation. Some examples are given in Table 3.

## 12. Conclusions

Electrospun nanocomposites/(nano)fibers (respectively electrospinning/electrospraying) require less amounts of raw materials, but properties are enhanced due to nanometric dimensions, which makes them a cost–effective alternative to conventional polymers and methods of packaging like Modified Atmosphere Packaging (MAP). Presently, encapsulation of bioactive compounds by electrospinning/electrospraying procedures is applied mainly at laboratory scale but also at pilot scale and industrially, with an impressive research interest with the aim of application in various domains as biomedical, biosensors, food preservation and safety, etc.

Although synthetic bioactive compounds are approved in many countries for food applications, they are not easily accepted by consumers, existing an increasing interest to replace them by natural bioactive compounds. The natural bioactive compounds can be used as food additives to preserve the food quality and safety, and as food supplements or nutraceuticals to correct nutritional deficiencies, maintain a suitable intake of nutrients, or to support physiological functions, etc. Bioactive compounds can be used as a single added component in a matrix or as blends of nanocomposites containing organic and inorganic bioactive compounds. Toxicological effects and specific regulation for the safety of human consumption and the environment are the main challenges in using most additives in food [254].

New classes of bioactive compounds are being developed such as cyanobacterial bioactive compounds, bioactive molecules from microalgae, nuisance cyanobacteria as anticancer agents, substances from fungi, and many other useful in therapeutic applications [243].

## Figures and Tables

**Table 1:** Main constituents of some EOs.

Essential Oil	Main Components	Observations	Ref.
Ginger, garlic, tick berry, and Mexican marigold	There were 18 major classes that were identified with average percent chemical composition of >1% with terpenes having the highest composition for all the tested plants. Other major chemical classes identified included esters, ketones, organosulfur compounds, alkanes, cycloalkanes, steroids, aromatic hydrocarbons, alkanols, cycloalkanols, alkenols, carbonates, fatty acids, carbaldehyde, aldehydes, alkenes, ethers, carboxylic acid, alkaloids, and organic acids	All the tested plants have similar chemical compounds and can therefore be exploited for synergistic utility.	[36]
*Aloe debrana* roots	A total of 14 compounds representing 99.1% of the EO composition isolated by hydrodistillation were identified with thymohydroquinone dimethyl ether (47.1%) as a major constituent. From simultaneous distillation extraction were identified 33 compounds, which represent 88.8% of the EO constituents with thymohydroquinone dimethyl ether (39.6%), thymol (7.7%), humulene epoxide II (5.8%), dibutyl phthalate (5.0%) and carvacrol (4.2%) as major components.	Antibacterial activity against Gram-positive *S. aureus* at 0.5 and 1.0 mg/mL. Antioxidant activity with IC50 values of 48 ± 12 micrograms/mL in 2,2-diphenyl-1-picrylhydrazyl (DPPH) and 51 ± 2 micrograms/mL in H_2_O_2_.	[37]
*Heteromorpha arborescens* leaves	The major constituents observed in the EO extracted by Solvent-Free Microwave Extraction include α-pinene (6%), D-limonene (11.27%), β–ocimene (9.09%), β–phellandrene (6.33%), β-mycene (8.49%), caryophyllene (5.96%), and camphene (4.28%). The main components obtained by hydrodistillation were α-pinene (4.41%), β-pinene (10.68%), β–ocimene (6.30%), germacrene-D (5.09%), humulene (5.55%), and α-elemene (6.18%).		[38]	
	α-pinene as the component (up to 75.40%) along with the other main components: eucalyptol, caryophyllene, borneol, camphene and verbenone.	Humidity and rainfalls did not affect EO components	[39]
Rosemary	The main constituents are camphor (5.0–21%), 1,8−cineole (15–55%), α-pinene (9.0–26%), borneol (1.5–5.0%), camphene (2.5–12%), b-pinene (2.0–9.0%) and limonene (1.5–5.0%) in proportions that vary according to the vegetative stage and bioclimatic conditions		[40,41]
Wild Populations of *Ferulago cassia Boiss*	The major components were chrysanthenyl acetate (13.54–24.49%), 2,3,6–trimethylbenzaldehyde (5.94–25.52%), L-limonene (4.69–27.44%), α-pinene (7.64–12.43%), β-myrcene (3.44–10.38%) and L-phellandrene (2.90–9.75%).		[42]
*Cryptocarya impressa*, *Cryptocarya infectoria*, and *Cryptocarya rugulosa*; three Cryptocarya species from Malaysia	High percentages of α-cadinol (40.7%) and 1,10-di-epi-cubenol (13.4%) in *C. impressa oil*, β-Caryophyllene (25.4%) and bicyclogermacrene (15.2%) in *C. infectoria* oil, while bicyclogermacrene (15.6%), δ-cadinene (13.8%), and α-copaene (12.3%) were predominate in *C. rugulosa* oil.		[43]
Fresh rhizomes, flowers; and leaves of *Zingiber kerrii Craib*	α–pinene; β-pinene; and terpinen–4–ol from the rhizome extract, (E)-caryophyllene from the flower extract, α-pinene; (E)-caryophyllene; and n-hexadecanoic acid from the leaf extract.	Due to the small amount of phenolic compounds, the EOs extracted from the rhizomes had low antioxidant activity and moderate activity against bacterial strains.	[44]
*Thymus convolutus Klokov*	camphor 16.6%.	Strong antimicrobial activity against *Escherichia coli*, *Enterobacter aerogenes*, *Proteus vulgaris*, and *Pseudomonas aeruginosa* with minimum inhibitory concentration (MIC) values of 125 micrograms/mL	[45]
Leaves and flowers of *Salvia hydrangea*	Oil composition was affected by the part of the plants used: the most abundant bioactives contained in leafs were (+)-spathulenol (16.07%), 1,8-cineole (13.96%), trans-caryophyllene (9.58%), β-pinene (8.91%) and β–eudesmol (5.33%) and those in flowers were caryophyllene oxide (35.47%), 1,8–cineole (9.54%), trans-caryophyllene (6.36%), β-eudesmol (4.11%), caryophyllenol-II (3.46%) and camphor (3.33%).	Both oils showed a significant inhibitory and lethal effect on the Gram-negative bacteria *Pseudomonas aeruginosa* (MIC ~ 16 µg/mL), *Shigella dysenteriae* and *Klebsiella pneumoniae* (MIC ~ 62 µg/mL).	[46]
Different Brazilian Celastraceae species	Cis- and trans-linalool oxide, nerylacetone, linalool, β-ionone, α-ionone, nerolidol, decanal, and dodecanoic acid.		[47]
fruit and herb of *Coriandrum sativum*	Commercial coriander EO is dominated by linalool (62.2–76.7%) with lesser quantities of α-pinene (0.3–11.4%), γ-terpinene (0.6–11.6%), and camphor (0.0–5.5%). Commercial cilantro essential oil is composed largely of (2E)-decenal (16.0–46.6%), linalool (11.8–29.8%), (2E)-decen-1-ol (0.0–24.7%), decanal (5.2–18.7%), (2E)-dodecenal (4.1–8.7%), and 1-decanol (0.0–9.5%).		[48]
*Curcuma longa*, *Pimenta dioica*, *Rosmarinus officinalis*, and *Syzygium aromaticum*	Eugenol (88% in *S. aromaticum* and 16% in *P. dioica*), methyl eugenol (53% in *P. dioica*), and α-turmerone (44%), β–turmerone (20%), and Ar-turmerone (17%) in *C. longa*. Major componets in *Rosmarinus officinalis* are 1,8-cineole (53%), α-pinene (15%), and (−) camphor (9%).	*S. aromaticum* EO exhibited the highest antifungal effect, followed by *P. dioica* and to a lesser extent *C. longa*. *Rosmarinus officinalis* poorly inhibited fungal growth.	[49]
*Liquidambar formosana*	(E)-caryophyllene (3.3–64.4%), α-pinene (0.6–34.5%), β-pinene (0.6–26.0%), camphene (0.3–17.3%), and limonene (0.2–7.9%). (-)-α-Pinene, (-)-β-pinene, (-)-camphene, and (-)-limonene were the dominant enantiomers.	Antimicrobial activities with MIC ≤ 625 micrograms/mL against a panel of potentially pathogenic bacteria.	[50]
Volatile compounds of the fruit and leaf EOs of the African star fruit, *Chrysophyllum albidum G. Don*		The fruit essential oil exhibited broad-spectrum antimicrobial activity in the antimicrobial susceptibility test, with MIC ranging from 0.195 to 6.250 mg/mL, while the leaf EOs showed antimicrobial activity with MIC in the range of 6.875–13.750 mg/mL.	[51]
Needles of *Pinus radiata D. Don*	monoterpene hydrocarbons (86.4%) with β-pinene (40.2%), limonene (25.5%) and α-pinene (15.2%).		[52]
Aerial parts of *Phlomis bucharica*, *P. salicifolia* and *P. sewerzowii*	thymol (20%) and camphor (14%) in *P. bucharica* oil. Methyl palmitate (51%) in *P. salicifolia* and thymol (35%) in *P. sewerzowii* essential oil.	The EOs of *P. salicifolia* showed the highest antibacterial activity.	[53]
Aerial parts of *Englerastrum gracillimum Th. C. E. Fries*	α-humulene (30.5%), followed by cubenol (19.8%), γ-muurolene (14.0%), (E)–β–caryophyllene (5.8%), β–gurjunene (5.2%), and curzerene (4.9%).	Antioxidant activity Antibacterial activity against multi-resistant *Acinetobacter baumannii* P1483, extended-spectrum β-lactamase (ESBL)-*Escherichia coli* Bu8566, *Salmonella spp*. H1548, *Proteus mirabilis* Bu190, *Enterobacter cloacae* Bu147, *Pseudomonas aeruginosa* (ATCC 27853), *Escherichia coli* (ATCC 25922), *Klebsiella pneumoniae* (ATCC 700603), methicillin-resistant *Staphylococcus aureus* P1123, *Enterococcus faecium* H3434, and *Staphylococcus aureus* (ATCC 25923).	[54]

**Table 2:** Antimicrobial/antioxidant electrospun nanofibers with applications in food preservation.

BC as EO and Other	Activity and Application	Ref.
Lemon essential oils	Lemon essential oil (LEO) was absorbed by thermally stable and porous vermiculite (VML) to form LEO/VML complex, which is further coupled with konjac glucomannan-grafted-poly (acrylic acid)/polyvinyl alcohol (KGM-g-PAA/PVA) electrospun composite. The VML (1 g) can significantly reduce LEO loss and achieves a sustained control LEO release from the electrospun composite, which can effectively inhibit the growth of *E. coli* during storage, thus prolonging shelf life of chilled pork meat for 3 days.	[139]
Rosemary	Rosemary essential oil was encapsulated in zein-electrospun fibers at different concentrations of loading (0%, 2.5%, 5%, and 10% *v*/*v*). Disc diffusion indicated that zein-electrospun mats generated inhibition zones against *S. aureus* and *E. coli*. The release test revealed that pH values significantly affect the release of rosemary essential oil from fibers. The results demonstrated how loading zein-electrospun fibers with rosemary essential oil can benefit food packaging.	[140]
Cinnamon	Electrospun polyvinyl alcohol/cinnamon essential oil/β-cyclodextrin (PVA/CEO/β-CD) antimicrobial nanofibrous film exhibited excellent antimicrobial activity S. aureus and E. coli.. Furthermore, the mild electrospinning process was favorable for maintaining greater cinnamon essential oil in the film resulting in an improved antimicrobial activity compared with that of casting film. The prolonged shelf-life of strawberries packed with the antimicrobial PVA/CEO/β–CD nano-film togheter with the preservation of the sensorial property during storage indicates its potential for the application in active food packaging. Additionally, it is non-toxic and biodegradable, thus potential in active food packaging for the concern of food security and environmental problems.	[141]
Thyme	Nanofibers based on poly(vinyl pyrrolidone)/gelatin/thyme essential oil using oil-in-water emulsions displayed good antibacterial activity against *S. aureus*, *E. coli*, *C. albicans*, *P. aeruginosa*, and *E. faecalis* increasing with thyme EO concentration. Nanofibers stored at 24 °C and 37 °C demonstrated antibacterial activity over a period of 192 h.	[142]
Lemongrass	Electrospun gelatin nanofibers with lemongrass essential oil (LEO) as potential biodegradable and active food packaging show antimicrobial activity against *Staphylococcus aureus* and *Salmonella Typhimurium*. Fourier transform infrared spectroscopy showed the effective penetration of LEO in gelatin fibers without chemical interaction or destroying the structure of LEO or gelatin. Thermal analyses indicated that thermal stability of the essential oil enhanced by encapsulation.	[143]
Oregano	Electrospun nanofibers prepared from poly(3-hydroxybutyrate-co-3-hydroxyvalerate) derived from fermented fruit waste (bio-papers) containing 2.5 wt% oregano essential oil and 2.25 wt% zinc oxide nanoparticles showed high antimicrobial activity for up to 48 days against *Staphylococcus aureus* and *Escherichia coli.*	[144]
Coconut sap (neera) from the coconut tree (*Cocos nucifera L.*) is a healthy and refreshing drink.Neera is highly susceptible to acetic natural fermentation process fermentation by the inherent yeasts (particularly *Saccharomyces cerevisiae*). These must be eliminated by filtration using polycaprolactone (PCL) membrane and so quality of the drink is preserved	Electrospun polycaprolactone nanofibrous membrane (mean thickness of 150 µm, 70% porosity, average fiber diameter 900 nm) was used for the filtration/removal of yeast from coconut neera (a natural drink that is rich in amino acids, polyphenols, vitamins, and minerals). The neera filtrate showed a 2 log-reduction in yeast load. The effective reusability of the membrane and stability of the nanofiber morphology at repeated usage was confirmed. The filtered coconut neera had significant changes in titratable acidity, pH, and color, slight reductions in the total polyphenolic content and minerals, while no significant changes were observed in total soluble solids content.	[145]

**Table 3:** Application of some EOs incorporated in films or coatings for food preservation and safety.

EO	Application	Ref.
Thyme and garlic	Mozzarella coated with the edible film (zein and +3% of a mixture of thyme and garlic essential oils 1:1). This film could be applied as natural additive, contributing to the microbiological and sensory characteristics of the mozzarella whith the benefit of 50% of salt reduction.	[244]
Tarragon	Tarragon essential oil was added to the sausages at a concentration that was reported to exhibit antimicrobial activity in meat (0.1% *v*/*w*). The 0.1% (*v*/*w*) addition of tarragon essential oil decreased the flavor and overall acceptability of the Frankfurter type sausages. However, it can be said that the undesired effect of the tarragon essential oil on the flavor and the overall acceptability could be eliminated by modifying the added amount of the essential oil.	[245]
Citrus	This study evaluated the antimicrobial potentials of Citrus EOs against spoilage microorganisms isolated from selected fruits. In vitro antimicrobial efficacies of Citrus EOs and their synergistic potentials were tested against isolated spoilage microorganisms (*Bacillus spp.*, *Micrococcus luteus*, *Serratia marcescens*, *Aspergillus spp.*, *Mucor piriformis*, *Fusarium oxysporum*, *Penicillium spp.*, *Rhizopus spp.*, *Alternaria alternata*). The synergism between lime and lemon at ratio 1:1 had better antimicrobial activity than each essential oil when used alone Gas chromatography–mass spectrometry (GC–MS) revealed the presence of limonene, beta-pinene, alpha-phellandrene, terpinen-4-ol, alpha-terpineol and geraniol in EOs of lime and lemon.	[246]
Juniper fruits (*Juniperus communis L.*), lemongrass leaves (*Cymbopogon citratus*), rosemary leaves (*Rosmarinus officinalis*), black pepper (*Piper nigrum*) fruits	*P. orientalis* strains isolated from food probes lose their ability to move, change their morphology, and also reduce their metabolic activity under the influence of oils in low concentrations. However, they do not die, and properties such as the ability to produce ammonia, the ability to production of indole from the amino acid tryptophan as well as the ability to assimilation of saccharides are maintained.	[247]
Rosemary alcholic extract as powder	Films of PLA, bio-plasticizers, vitamin E and rosehip seed oil encapsulated into chitosan by the emulsion method were obtained by melt compounding to obtain a controlable composition for films with different properties/antimicrobial and antioxidant activity,	[13]
Oregano and thyme	In vitro studies have shown that oregano and thyme EOs are effective against foodborne bacteria, isolated from fermented meat products and cheeses, such as *Escherichia coli*, *Listeria monocytogenes*, *Salmonella spp.*, and *Staphylococcus aureus*. However, EOs of thyme and oregano seem to control the growth of fungi *Botrytis cinerea* and *Aspergillus spp.*, affecting the shelf-life of fruits during postharvest. The EOs of sage and rosemary have shown little or no antimicrobial activity. The optimal composition used for shelf-life studies was determined based on the results of in vitro studies. Shelf-life extension studies using several EOs (cinnamon, clove, oregano, rosemary, sage, and thyme) and aromatic and medicinal plants were performed using pork meat, goat cheese, strawberries, and table grapes. Practical applications. For shelf-life studies a cotton gauze impregnated with 1:10 EO dilution was put (a) inside the polyethyleneterephthalate (PET) boxes containing strawberries or grapes (EO diluted in food grade ethanol) or (b) on the inner surface of PET boxes containing the meat (EO diluted in water). For cheese shelf-life studies oregano EO as well as oregano dry leaves were used as ingredients in cheese production. The use of cinnamon, sage, and thyme EOs in the preservation of strawberries and table grapes enabled the extension of shelf−life by controlling the fungal growth. The use of the oregano and oregano EO toghether enabled the extension of the shelf-life for cheese from 6 to 8 days, since no significant changes were observed in the microbiota of chees. Due to its in vitro antibacterial activity against gram−negative bacteria thyme EO was effective in controlling the population of enterobacteria present in pork meat.	[248]
Thyme and clove	The addition of thyme and clove essential oils (especially thyme) to the sausage composition increased the shelf-life (frozen storage-18°C for three months), prevented the deterioration of sausage, inhibited lipid peroxidation and decreased the necessary nitrite’s proportion to sausage for avoiding the formation of carcinogenic N-nitrosamines, lowered residual nitrite, and TBC (Total Bacteria Count) in sausage. The tested EOs increased the inhibitory influence of nitrite on Proteus, Klebsiella, *Aspergillus niger*, and *Candida albicans* in sausage.	[249]
Argan and clove	PLA coated with chitosan/argan or clove EO showed increased hydrophilicity, especially for argan, in retarding the food spoilage of meat, and cheese. Argan, and clove oil offered good UV protection, biodegradability of PLA films with the antibacterial/antioxidant function of vegetal oils.	[250]
Cardamom, cinnamon, clove, eucalyptus, lemongrass, lime, nutmeg and rosemary	In vitro tests of the essential oils were evaluated for their antimicrobial activity against three *Pseudomonas* species associated with microbial spoilage of refrigerated tilapia. Cinnamon EO had the highest antimicrobial activity, followed by clove EO. The remaining essential oils had weak activity. The Cinnamon EO reduced the *Pseudomonas* viable count in in fish extract model at refrigeration temperature but to a lesser extent than when it was applied at the same concentrations in culture medium.	[251]
Cinnamon	The antifungal activity against *A. niger* and *M. racemous* were improved by the addition of nano and micro emulsion of cinnamon EO to the carboxymethyl cellulose edible films.	[252]
Thyme, lemongrass, juniper, oregano, sage, fennel, rosemary, mint, rosehips, dill	Antimicrobial activity of different selected EOs on some pathogenic and spoilage bacteria isolated from the surface of various fresh vegetables. The most resistant isolates appeared to be *Curtobacterium herbarum*, *Achromobacter xylosoxidans*, and *Enterobacter ludwigii*, while *Pseudomonas hibiscicola* was the most sensitive. Of the chosen plant essential oils, the most pronounced antimicrobial effect was detected in the case of oregano. The essential oils of thyme and mint also showed elevated antimicrobial activity. A synergistic effect was observed in case of five combinations of essential oil therefore thay are good candidates for the preservation of fresh vegetables.	[253]

## Data Availability

No supporting data available.

## References

[1] Yildirim S., Röcker B., Pettersen M.K., Nilsen-Nygaard J., Ayhan Z., Rutkaite R., Radusin T., Suminska P., Marcos B., Coma V. (2018). Active packaging applications for food. Compr. Rev. Food Sci. Food Saf..

[2] Papadochristopoulos A., Kerry J., Fegan N., Burgess C., Duffy G. (2021). Natural Anti-Microbials for Enhanced Microbial Safety and Shelf-Life of Processed Packaged Meat. Foods.

[3] Liguori G., Sortino G., Gullo G., Inglese P. (2021). Effects of Modified Atmosphere Packaging and Chitosan Treatment on Quality and Sensorial Parameters of Minimally Processed cv. ‘Italia’ Table Grapes. Agronomy.

[4] Salmieri S., Islam F., Khan R.A., Hossain F.M., Ibrahim H., Miao C., Hamad W.Y., Lacroix M. (2014). Antimicrobial nanocomposite films made of poly(*Lactic acid*)–cellulose nanocrystals (PLA–CNC) in food applications—part B: Effect of oregano essential oil release on the inactivation of Listeria monocytogenes in mixed vegetables. Cellulose.

[5] Dumitriu R.P., Mitchell G.R., Davis F.J., Vasile C. (2017). Functionalized Coatings by Electrospinning for Anti-oxidant Food Packaging. Procedia Manuf..

[6] Chen C., Xu Z., Ma Y., Liu J., Zhang Q., Tang Z., Fu K., Yang F., Xie J. (2018). Properties, vapour-phase antimicrobial and antioxidant activities of active poly (*Vinyl alcohol*) packaging films incorporated with clove oil. Food Control.

[7] Fang Z., Zhao Y., Warner R.D., Johnson S. (2017). Active and intelligent packaging in meat industry. Trends Food Sci. Technol..

[8] Kwon S.-J., Chang Y., Han J. (2017). Oregano essential oil-based natural antimicrobial packaging film to inactivate Salmonella enterica and yeasts/molds in the atmosphere surrounding cherry tomatoes. Food Microbiol..

[9] Munteanu S.B., Vasile C. (2019). Vegetable Additives in Food Packaging Polymeric Materials. Polymers.

[10] Vasile C., Baican M. (2021). Progresses in Food Packaging, Food Quality, and Safety—Controlled-Release Antioxidant and/or Antimicrobial Packaging. Molecules.

[11] Wan Z.-L., Guo J., Yang X.-Q. (2015). Plant protein-based delivery systems for bioactive ingredients in foods. Food Funct..

[12] Vasile C., Stoleru E., Darie-Niţa R.N., Dumitriu R.P., Pamfil D., Tarţau L. (2019). Biocompatible materials based on plasticized Poly (*Lactic acid*), chitosan and rosemary ethanolic Extract I. Effect of chitosan on the properties of plasticized Poly (*Lactic acid*) materials. Polymers.

[13] Darie-Niță R., Râpă M., Sivertsvik M., Rosnes J., Popa E., Dumitriu R., Marincaș O., Matei E., Predescu C., Vasile C. (2021). PLA-Based Materials Containing Bio-Plasticizers and Chitosan Modified with Rosehip Seed Oil for Ecological Packaging. Polymers.

[14] Oliver-Ortega H., Vandemoortele V., Bala A., Julian F., Méndez J.A., Espinach F.X. (2021). Nanoclay Effect into the Biodegradation and Processability of Poly(*Lactic acid*) Nanocomposites for Food Packaging. Polymers.

[15] Maurya A., Prasad J., Das S., Dwivedy A.K. (2021). Essential Oils and Their Application in Food Safety. Front. Sustain. Food Syst..

[16] Taghavi T., Kim C., Rahemi A. (2018). Role of Natural Volatiles and Essential Oils in Extending Shelf Life and Controlling Postharvest Microorganisms of Small Fruits. Microorganisms.

[17] Baptista-Silva S., Borges S., Ramos O.L., Pintado M., Sarmento B. (2020). The progress of essential oils as potential therapeutic agents: A review. J. Essent. Oil Res..

[18] Sharifi-Rad J., Sureda A., Tenore G.C., Daglia M., Sharifi-Rad M., Valussi M., Tundis R., Sharifi-Rad M., Loizzo M.R., Ademiluyi A.O. (2017). Biological activities of essential oils: From plant chemoecology to traditional healing systems. Molecules.

[19] Dhifi W., Bellili S., Jazi S., Bahloul N., Mnif W. (2016). Essential Oils’ Chemical Characterization and Investigation of Some Biological Activities: A Critical Review. Medicines.

[20] Banasaz S., Morozova K., Ferrentino G., Scampicchio M. (2020). Encapsulation of Lipid-Soluble Bioactives by Nanoemulsions. Molecules.

[21] Vuko E., Dunkić V., Ruščić M., Nazlić M., Mandić N., Soldo B., Šprung M., Fredotović Ž. (2021). Chemical Composition and New Biological Activities of Essential Oil and Hydrosol of Hypericum perforatum L. ssp. veronense (Schrank) H. Lindb. Plants.

[22] Sharmeen J., Mahomoodally F., Zengin G., Maggi F. (2021). Essential Oils as Natural Sources of Fragrance Compounds for Cosmetics and Cosmeceuticals. Molecules.

[23] Bhavaniramya S., Vishnupriya S., Al-Aboody M.S., Vijayakumar R., Baskaran D. (2019). Role of essential oils in food safety: Antimicrobial and antioxidant applications. Grain Oil Sci. Technol..

[24] Pandey A.K., Kumar P., Singh P., Tripathi N.N., Bajpai V.K. (2017). Essential Oils: Sources of Antimicrobials and Food Preservatives. Front. Microbiol..

[25] Vasile C., Sivertsvik M., Mitelut A.C., Brebu M.A., Stoleru E., Rosnes J.T., Tănase E.E., Khan W., Pamfil D., Cornea C.P. (2017). Comparative Analysis of the Composition and Active Property Evaluation of Certain Essential Oils to Assess their Potential Applications in Active Food Packaging. Materials.

[26] Wu Z., Tan B., Liu Y., Dunn J., Guerola P.M., Tortajada M., Cao Z., Ji P. (2019). Chemical Composition and Antioxidant Properties of Essential Oils from Peppermint, Native Spearmint and Scotch Spearmint. Molecules.

[27] Gutiérrez-Del-Río I., López-Ibáñez S., Magadán-Corpas P., Fernández-Calleja L., Pérez-Valero Á., Tuñón-Granda M., Miguélez E., Villar C., Lombó F. (2021). Terpenoids and Polyphenols as Natural Antioxidant Agents in Food Preservation. Antioxidants.

[28] Cardoso-Ugarte G.A., Sosa-Morales M.E. (2021). Essential Oils from Herbs and Spices as Natural Antioxidants: Diversity of Promising Food Applications in the past Decade. Food Rev. Int..

[29] Amorati R., Foti M.C., Valgimigli L. (2013). Antioxidant activity of essential oils. J. Agric. Food Chem..

[30] Čabarkapa I., Čolović R., Đuragić O., Popović S., Kokić B., Milanov D., Pezo L. (2019). Anti-biofilm activities of essential oils rich in carvacrol and thymol against Salmonella enteritidis. Biofouling.

[31] Guimarães A.C., Meireles L.M., Lemos M.F., Guimarães M.C.C., Endringer D.C., Fronza M., Scherer R. (2019). Antibacterial Activity of Terpenes and Terpenoids Present in Essential Oils. Molecules.

[32] Swamy M.K., Akhtar M.S., Sinniah U.R. (2016). Antimicrobial Properties of Plant Essential Oils against Human Pathogens and Their Mode of Action: An Updated Review. Evid. Based Complement. Altern. Med..

[33] Mannu A., Melito S., Petretto G.L., Manconi P., Pintore G.M., Chessa M. (2020). Geographical variation of the chemical composition in essential oils extracted from Sardinian Salvia verbenaca. Nat. Prod. Res..

[34] Sarmoum R., Haid S., Biche M., Djazouli Z., Zebib B., Merah O. (2019). Effect of Salinity and Water Stress on the Essential Oil Components of Rosemary (*Rosmarinus officinalis* L.). Agronomy.

[35] Aćimović M., Pezo L., Zeremski T., Lončar B., Jeromela A.M., Jeremic J.S., Cvetković M., Sikora V., Ignjatov M. (2021). Weather Conditions Influence on Hyssop Essential Oil Quality. Processes.

[36] Mugao L.G., Gichimu B.M., Muturi P.W., Mukono S.T. (2020). Characterization of the Volatile Components of Essential Oils of Selected Plants in Kenya. Biochem. Res. Int..

[37] Getahun T., Sharma V., Gupta N. (2020). Chemical Composition and Biological Activity of Essential Oils from Aloe debrana roots. J. Essent. Oil Bear. Plants.

[38] Abifarin T.O., Otunola G.A., Afolayan A.J. (2020). Chemical Composition of Essential Oils Obtained from Heteromorpha arborescens (Spreng.) Cham. and Schltdl Leaves Using Two Extraction Methods. Sci. World J..

[39] Serralutzu F., Stangoni A., Amadou B., Tijan D., Re G.A., Marceddu S., Dore A., Bullitta S. (2020). Essential oil composition and yield of a Rosmarinus officinalis L. natural population with an extended flowering season in a coastal Mediterranean environment and perspectives for exploitations. Genet. Resour. Crop. Evol..

[40] Andrade J.M., Faustino C., Garcia C., Ladeiras D., Reis C.P., Rijo P. (2018). Rosmarinus officinalis L.: An update review of its phytochemistry and biological activity. Futur. Sci. OA.

[41] Darie-Niţă R.N., Vasile C., Stoleru E., Pamfil D., Zaharescu T., Tarţău L., Tudorachi N., Brebu M.A., Pricope G.M., Dumitriu R.P. (2018). Evaluation of the Rosemary Extract Effect on the Properties of Polylactic Acid-Based Materials. Materials.

[42] Sanli A., Karadogan T., Tosun B., Erbas S. (2020). Variation of Chemical Composition of Essential Oils in Wild Populations of Ferulago cassia Boiss. From Turkey. J. Essent. Oil Bear. Plants.

[43] Azhar M.A., Salleh W.M., Khamis S. (2020). Essential oil composition of three Cryptocarya species from Malaysia. Z. Für Nat. C.

[44] Pintatum A., Laphookhieo S., Logie E., Berghe W.V., Maneerat W. (2020). Chemical Composition of Essential Oils from Different Parts of Zingiber kerrii Craib and Their Antibacterial, Antioxidant, and Tyrosinase Inhibitory Activities. Biomolecules.

[45] Yuceturk S.C., Turkoglu S.A., Kockar F., Kucukbay F.Z., Azaz A.D. (2021). Essential oil chemical compositionvirgula antimicrobialvirgula anticancervirgula and antioxidant effects of Thymus convolutus Klokov in Turkey. Z. Für Nat. C.

[46] Ghavam M., Manca M.L., Manconi M., Bacchetta G. (2020). Chemical composition and antimicrobial activity of essential oils obtained from leaves and flowers of Salvia hydrangea DC. ex Benth. Sci. Rep..

[47] Camargo K.C., Duarte L.P., Vidal D.M., Pereira H.V., Pereira R.C.G., De Aguilar M.G., De Sousa G.F., Filho S.A.V., Mercadante-Simões M.O., Messias M.C.T.B. (2020). Chemodiversity of Essential Oils from Nine Species of Celastraceae. Chem. Biodivers..

[48] Satyal P., Setzer W.N. (2020). Chemical Compositions of Commercial Essential Oils From Coriandrum sativum Fruits and Aerial Parts. Nat. Prod. Commun..

[49] Achimón F., Brito V.D., Pizzolitto R.P., Sanchez A.R., Gómez E.A., Zygadlo J.A. (2021). Chemical composition and antifungal properties of commercial essential oils against the maize phytopathogenic fungus Fusarium verticillioides. Rev. Argent. Microbiol..

[50] Decarlo A., Zeng T., Dosoky N.S., Satyal P., Setzer W.N. (2020). The Essential Oil Composition and Antimicrobial Activity of Liquidambar formosana Oleoresin. Plants.

[51] Nartey D., Gyesi J.N., Borquaye L.S. (2021). Chemical Composition and Biological Activities of the Essential Oils of Chrysophyllum albidum G. Don (African Star Apple). Biochem. Res. Int..

[52] Ismail A., Habiba K., Yassine M., Mohsen H., Bassem J., Lamia H. (2021). Essential oils of Tunisian Pinus radiata D. Donvirgula chemical composition and study of their herbicidal activity. Vietnam J. Chem..

[53] Mamadalieva N.Z., Youssef F.S., Ashour M.L., Akramov D.K., Sasmakov S.A., Ramazonov N.S., Azimova S.S. (2021). A comparative study on chemical composition and antimicrobial activity of essential oils from three Phlomis species from Uzbekistan. Nat. Prod. Res..

[54] Abba B.N., Ilagouma A.T., Amadou I., Romane A. (2021). Chemical Profiling, Antioxidant and Antibacterial Activities of Essential Oil From Englerastrum gracillimum Th. C. E. Fries Growing in Niger. Nat. Prod. Commun..

[55] Rao J., Chen B., Mc Clements D.J. (2019). Improving the Efficacy of Essential Oils as Antimicrobials in Foods: Mechanisms of Action. Annu. Rev. Food Sci. Technol..

[56] Kyriakoudi A., Spanidi E., Mourtzinos I., Gardikis K. (2021). Innovative Delivery Systems Loaded with Plant Bioactive Ingredients: Formulation Approaches and Applications. Plants.

[57] Rehman A., Jafari S.M., Aadil R.M., Assadpour E., Randhawa M.A., Mahmood S. (2020). Development of active food packaging via incorporation of biopolymeric nanocarriers containing essential oils. Trends Food Sci. Technol..

[58] Ghareaghajlou N., Hallaj-Nezhadi S., Ghasempour Z. (2021). Red cabbage anthocyanins: Stability, extraction, biological activities and applications in food systems. Food Chem..

[59] Zhuang S., Li Y., Jia S., Hong H., Liu Y., Luo Y. (2019). Effects of pomegranate peel extract on quality and microbiota composition of bighead carp (*Aristichthys nobilis*) fillets during chilled storage. Food Microbiol..

[60] Chambre D.R., Moisa C., Lupitu A., Copolovici L., Pop G., Copolovici D.-M. (2020). Chemical composition, antioxidant capacity, and thermal behavior of Satureja hortensis essential oil. Sci. Rep..

[61] Pavoni L., Perinelli D.R., Bonacucina G., Cespi M., Palmieri G.F. (2020). An Overview of Micro- and Nanoemulsions as Vehicles for Essential Oils: Formulation, Preparation and Stability. Nanomaterials.

[62] Liao W., Badri W., Dumas E., Ghnimi S., Elaissari A., Saurel R., Gharsallaoui A. (2021). Nanoencapsulation of Essential Oils as Natural Food Antimicrobial Agents: An Overview. Appl. Sci..

[63] Carpena M., Nuñez-Estevez B., Soria-Lopez A., Garcia-Oliveira P., Prieto M.A. (2021). Essential Oils and Their Application on Active Packaging Systems: A Review. Resources.

[64] Mucha W., Witkowska D. (2021). The Applicability of Essential Oils in Different Stages of Production of Animal-Based Foods. Molecules.

[65] Mohammadalinejhad S., Kurek M. (2021). Microencapsulation of Anthocyanins—Critical Review of Techniques and Wall Materials. Appl. Sci..

[66] Ali A., Chen Y., Liu H., Yu L., Baloch Z., Khalid S., Zhu J., Chen L. (2019). Starch-based antimicrobial films functionalized by pomegranate peel. Int. J. Biol. Macromol..

[67] Beltrán Sanahuja A., Valdés García A. (2021). New Trends in the Use of Volatile Compounds in Food Packaging. Polymers.

[68] Pateiro M., Gómez B., Munekata P., Barba F., Putnik P., Kovačević D., Lorenzo J. (2021). Nanoencapsulation of Promising Bioactive Compounds to Improve Their Absorption, Stability, Functionality and the Appearance of the Final Food Products. Molecules.

[69] He L., Lan W., Ahmed S., Qin W., Liu Y. (2019). Electrospun polyvinyl alcohol film containing pomegranate peel extract and sodium dehydroacetate for use as food packaging. Food Packag. Shelf Life.

[70] Serrano-Casas V., Pérez-Chabela M.L., Cortés-Barberena E., Totosaus A. (2017). Improvement of lactic acid bacteria viability in acid conditions employing agroindustrial co-products as prebiotic on alginate ionotropic gel matrix co-encapsulation. J. Funct. Foods.

[71] Lin L., Zhu Y., Cui H. (2018). Electrospun thyme essential oil/gelatin nanofibers for active packaging against Campylobacter jejuni in chicken. LWT.

[72] Fonseca L.M., Cruxen C.E.D.S., Bruni G.P., Fiorentini Â.M., Zavareze E.D.R., Lim L.-T., Dias A.R.G. (2019). Development of antimicrobial and antioxidant electrospun soluble potato starch nanofibers loaded with carvacrol. Int. J. Biol. Macromol..

[73] López de Dicastillo C., López-Carballo G., Gavara R., Muriel Galet V., Guarda A., Galotto M.J. (2019). Improving polyphenolic thermal stability of Aristotelia Chilensis fruit extract by encapsulation within electrospun cyclodextrin capsules. J. Food Process. Preserv..

[74] Saucedo-Zuñiga J., Sánchez-Valdes S., Ramírez-Vargas E., Guillen L., Ramos-Devalle L., Graciano-Verdugo A., Uribe-Calderón J., Valera-Zaragoza M., Lozano-Ramírez T., Rodríguez-González J. (2021). Controlled release of essential oils using laminar nanoclay and porous halloysite/essential oil composites in a multilayer film reservoir. Microporous Mesoporous Mater..

[75] Bastos L.P.H., Vicente J., dos Santos C.H.C., de Carvalho M.G., Garcia-Rojas E.E. (2020). Encapsulation of black pepper (*Piper nigrum L*.) essential oil with gelatin and sodium alginate by complex coacervation. Food Hydrocoll..

[76] Kumar S., Mukherjee A., Dutta J. (2020). Chitosan based nanocomposite films and coatings: Emerging antimicrobial food packaging alternatives. Trends Food Sci. Technol..

[77] Munir S., Hu Y., Liu Y., Xiong S. (2019). Enhanced properties of silver carp surimi-based edible films incorporated with pomegranate peel and grape seed extracts under acidic condition. Food Packag. Shelf Life.

[78] Stoleru E., Dumitriu R.P., Brebu M., Vasile C., Enache A. (2020). Development of Bioactive Polymeric Materials by Incorporation of Essential/Vegetal Oils into Biopolymer Matrices. Proceedings.

[79] Nguyen T.T.T., Le T.V.A., Dang N.N., Nguyen D.C., Nguyen P.T.N., Tran T.T., Nguyen Q.V., Bach L.G., Pham T.D.T.N. (2021). Microencapsulation of Essential Oils by Spray-Drying and Influencing Factors. J. Food Qual..

[80] Lammari N., Louaer O., Meniai A.H., Elaissari A. (2020). Encapsulation of Essential Oils via Nanoprecipitation Process: Overview, Progress, Challenges and Prospects. Pharmaceutics.

[81] Celebioglu A., Uyar T. (2021). Electrohydrodynamic encapsulation of eugenol-cyclodextrin complexes in pullulan nanofibers. Food Hydrocoll..

[82] de Souza E.J.D., Kringel D.H., Dias A.R.G., Zavareze E.D.R. (2021). Polysaccharides as wall material for the encapsulation of essential oils by electrospun technique. Carbohydr. Polym..

[83] Ibili H., Dasdemir M., Çankaya İ., İrem T., Orhan M., Güneşoğlu C., Anul S.A. (2021). Investigation of poly(*Lactic acid*) nanocapsules containing the plant extract via coaxial electrospraying method for functional nonwoven applications. J. Ind. Text..

[84] Munteanu B.S., Sacarescu L., Vasiliu A.-L., Hitruc G.E., Pricope G.M., Sivertsvik M., Rosnes J.T., Vasile C. (2018). Antioxidant/Antibacterial Electrospun Nanocoatings Applied onto PLA Films. Materials.

[85] Estevez-Areco S., Guz L., Candal R., Goyanes S. (2018). Release kinetics of rosemary (*Rosmarinus officinalis*) polyphenols from polyvinyl alcohol (PVA) electrospun nanofibers in several food simulants. Food Packag. Shelf Life.

[86] Fabra M.J., López-Rubio A., Lagaron J.M. (2016). Use of the electrohydrodynamic process to develop active/bioactive bilayer films for food packaging applications. Food Hydrocoll..

[87] Lin L., Mao X., Sun Y., Rajivgandhi G., Cui H. (2019). Antibacterial properties of nanofibers containing chrysanthemum essential oil and their application as beef packaging. Int. J. Food Microbiol..

[88] Bruni G.P., de Oliveira J.P., Gómez-Mascaraque L.G., Fabra M.J., Martins V.G., da Rosa Zavareze E., López-Rubio A. (2020). Electrospun β-carotene–loaded SPI: PVA fiber mats produced by emulsion-electrospinning as bioactive coatings for food packaging. Food Packag. Shelf Life.

[89] Subrahmanya T.M., Arshad A.B., Lin P.T., Widakdo J., Makari H.K., Austria H.F., Hu C.C., Lai J.Y., Hung W.S. (2021). A review of recent progress in polymeric electrospun nanofiber membranes in addressing safe water global issues. RSC Adv..

[90] Rostami M.R., Yousefi M., Khezerlou A., Mohammadi M.A., Jafari S.M. (2019). Application of different biopolymers for nanoencapsulation of antioxidants via electrohydrodynamic processes. Food Hydrocoll..

[91] Costa L.M.M., Bretas R.E.S., Gregorio R. (2010). Effect of Solution Concentration on the Electrospray/Electrospinning Transition and on the Crystalline Phase of PVDF. Mater. Sci. Appl..

[92] Partheniadis I., Nikolakakis I., Laidmäe I., Heinämäki J. (2020). A mini-review: Needleless electrospinning of nanofibers for pharmaceutical and biomedical applications. Processes.

[93] Prabu G.T.V., Dhurai B. (2020). A Novel Profiled Multi-Pin Electrospinning System for Nanofiber Production and Encapsulation of Nanoparticles into Nanofibers. Sci. Rep..

[94] Karpińska A., Simaite A., Buzgo M. (2020). Theoretical Models of the Most Promising Needle-Free Electrospinning Systems for Drug Delivery Applications. Proceedings.

[95] Zare M., Dziemidowicz K., Williams G., Ramakrishna S. (2021). Encapsulation of Pharmaceutical and Nutraceutical Active Ingredients Using Electrospinning Processes. Nanomaterials.

[96] Mc Clellan P., Landis W.J. (2016). Recent Applications of Coaxial and Emulsion Electrospinning Methods in the Field of Tissue Engineering. BioResearch Open Access.

[97] Faki R., Gursoy O., Yilmaz Y. (2019). Effect of Electrospinning Process on Total Antioxidant Activity of Electrospun Nanofibers Containing Grape Seed Extract. Open Chem..

[98] Zhang C., Li Y., Wang P., Zhang H. (2020). Electrospinning of nanofibers: Potentials and perspectives for active food packaging. Compr. Rev. Food Sci. Food Saf..

[99] Rezaei A., Fathi M., Jafari S.M. (2019). Nanoencapsulation of hydrophobic and low-soluble food bioactive compounds within different nanocarriers. Food Hydrocoll..

[100] Wen P., Zong M.-H., Linhardt R.J., Feng K., Wu H. (2017). Electrospinning: A novel nano-encapsulation approach for bioactive compounds. Trends Food Sci. Technol..

[101] Fang Z., Bhandari B. (2010). Encapsulation of polyphenols—A review. Trends Food Sci. Technol..

[102] Amjadi S., Almasi H., Ghorbani M., Ramazani S. (2020). Reinforced ZnONPs/rosemary essential oil-incorporated zein electrospun nanofibers by κ-carrageenan. Carbohydr. Polym..

[103] Aytac Z., Ipek S., Durgun E., Tekinay T., Uyar T. (2017). Antibacterial electrospun zein nanofibrous web encapsulating thymol/cyclodextrin-inclusion complex for food packaging. Food Chem..

[104] Rostami M., Ghorbani M., Mohammadi M.A., Delavar M., Tabibiazar M., Ramezani S. (2019). Development of resveratrol loaded chitosan-gellan nanofiber as a novel gastrointestinal delivery system. Int. J. Biol. Macromol..

[105] Charpashlo E., Ghorani B. (2021). Mohebbi, Mber structures. Food Hydrocoll..

[106] Rodríguez-Sánchez I.J., Vergara-Villa N.F., Clavijo-Grimaldo D., Fuenmayor C.A., Zuluaga-Domínguez C.M. (2020). Ultra. Multilayered electrospinning strategy for increasing the bioaccessibility of lycopene in gelatin-based sub-micron fi thin single and multiple layer electrospun fibrous membranes of polycaprolactone and polysaccharides. J. Bioact. Compat. Polym..

[107] Balik B.A., Argin S., Lagaron J.M., Torres-Giner S. (2019). Preparation and Characterization of Electrospun Pectin-Based Films and Their Application in Sustainable Aroma Barrier Multilayer Packaging. Appl. Sci..

[108] Pardo-Figuerez M., López-Córdoba A., Torres-Giner S., Lagaron J.M. (2018). Superhydrophobic Bio-Coating Made by Co-Continuous Electrospinning and Electrospraying on Polyethylene Terephthalate Films Proposed as Easy Emptying Transparent Food Packaging. Coatings.

[109] Figueroa-Lopez K.J., Cabedo L., Lagaron J.M., Torres-Giner S. (2020). Development of Electrospun Poly(3-hydroxybutyrate-co-3-hydroxyvalerate) Monolayers Containing Eugenol and Their Application in Multilayer Antimicrobial Food Packaging. Front. Nutr..

[110] Atarés L., Chiralt A. (2016). Essential oils as additives in biodegradable films and coatings for active food packaging. Trends Food Sci. Technol..

[111] Nguyen P., Vo T., Tran T., Le T., Mai H., Long G. (2020). Encapsulation efficiency and thermal stability of lemongrass (*Cymbopogon citratus*) essential oil microencapsulated by the spray-drying process. Food Res..

[112] Tavassoli-Kafrani E., Goli S.A.H., Fathi M. (2018). Encapsulation of Orange Essential Oil Using Cross-linked Electrospun Gelatin Nanofibers. Food Bioprocess Technol..

[113] Göksen G., Fabra M.J., Ekiz H.I., López-Rubio A. (2020). Phytochemical-loaded electrospun nanofibers as novel active edible films: Characterization and antibacterial efficiency in cheese slices. Food Control.

[114] Zhang Y., Zhang Y., Zhu Z., Jiao X., Shang Y., Wen Y. (2019). Encapsulation of Thymol in Biodegradable Nanofiber via Coaxial Eletrospinning and Applications in Fruit Preservation. J. Agric. Food Chem..

[115] Aydogdu A., Sumnu G., Sahin S. (2019). Fabrication of gallic acid loaded Hydroxypropyl methylcellulose nanofibers by electrospinning technique as active packaging material. Carbohydr. Polym..

[116] Fonseca L.M., De Oliveira J.P., Crizel R.L., Da Silva F.T., Zavareze E.D.R., Borges C.D. (2020). Electrospun Starch Fibers Loaded with Pinhão (*Araucaria angustifolia*) Coat Extract Rich in Phenolic Compounds. Food Biophys..

[117] Fonseca L.M., Radünz M., Hackbart H.C.D.S., Da Silva F.T., Camargo T.M., Bruni G.P., Monks J.L.F., Zavareze E.D.R., Dias A.R.G. (2020). Electrospun potato starch nanofibers for thyme essential oil encapsulation: Antioxidant activity and thermal resistance. J. Sci. Food Agric..

[118] Shetta A., Kegere J., Mamdouh W. (2019). Comparative study of encapsulated peppermint and green tea essential oils in chitosan nanoparticles: Encapsulationvirgula thermal stabilityvirgula in-vitro releasevirgula antioxidant and antibacterial activities. Int. J. Biol. Macromol..

[119] Yeh H.-F., Luo C.-Y., Lin C.-Y., Cheng S.-S., Hsu Y.-R., Chang S.-T. (2013). Methods for Thermal Stability Enhancement of Leaf Essential Oils and Their Main Constituents from Indigenous Cinnamon (*Cinnamomum osmophloeum*). J. Agric. Food Chem..

[120] Da Rosa C.G., Maciel M.V.D.O.B., de Carvalho S.M., de Melo A.P.Z., Jummes B., da Silva T., Martelli S.M., Villetti M.A., Bertoldi F.C., Barreto P.L.M. (2015). Characterization and evaluation of physicochemical and antimicrobial properties of zein nanoparticles loaded with phenolics monoterpenes. Colloids Surf. A Physicochem. Eng. Asp..

[121] Vasile C., Tudorachi N., Zaharescu T., Darie-Nita R.N., Cheaburu-Yilmaz C.N. (2020). Study on Thermal Behavior of Some Biocompatible and Biodegradable Materials Based on Plasticized PLAvirgula Chitosanvirgula and Rosemary Ethanolic Extract. Int. J. Polym. Sci..

[122] Zou Y., Zhang C., Wang P., Zhang Y., Zhang H. (2020). Electrospun chitosan/polycaprolactone nanofibers containing chlorogenic acid-loaded halloysite nanotube for active food packaging. Carbohydr. Polym..

[123] De Silva R.T., Dissanayake R.K., Mantilaka M.M.M.G.P.G., Wijesinghe W.P.S.L., Kaleel S.S., Premachandra T.N., Weerasinghe L., Amaratunga G.A.J., de Silva K.M.N. (2018). Drug-Loaded Halloysite Nanotube-Reinforced Electrospun Alginate-Based Nanofibrous Scaffolds with Sustained Antimicrobial Protection. ACS Appl. Mater. Interfaces.

[124] Porto M.D.A., Fonseca L.M., Da Silva F.T., Bruni G.P., Zavareze E.D.R., Dias A.R.G. (2020). Crosslinked electrospun polyvinyl alcohol-based containing immobilized α-amilase for food application. J. Food Process. Preserv..

[125] Moreno-Cortez I.E., Romero-García J., Gonzàlez V., García-Gutierrez D.I., Garza-Navarro M.A., Cruz-Silva R. (2015). Encapsulation and immobilization of papain in electrospun nanofibrous membranes of PVA cross-linked with glutaraldehyde vapor. Mater. Sci. Eng. C.

[126] Brahmi F., Abdenour A., Bruno M., Silvia P., Alessandra P., Danilo F., Drifa Y.-G., Fahmi E.M., Khodir M., Mohamed C. (2016). Chemical composition and in vitro antimicrobial, insecticidal and antioxidant activities of the essential oils of *Mentha pulegium* L. and *Mentha rotundifolia* (L.) Huds growing in Algeria. Ind. Crop. Prod..

[127] Yen H.-F., Hsieh C.-T., Hsieh T.-J., Chang F.-R., Wang C.-K. (2015). In vitro anti-diabetic effect and chemical component analysis of 29 essential oils products. J. Food Drug Anal..

[128] Andrade M.A., Ribeiro-Santos R., Bonito M.C.C., Saraiva M., Sanches-Silva A. (2018). Characterization of rosemary and thyme extracts for incorporation into a whey protein based film. LWT.

[129] López-Córdoba A., Medina-Jaramillo C., Piñeros-Hernandez D., Goyanes S. (2017). Cassava starch films containing rosemary nanoparticles produced by solvent displacement method. Food Hydrocoll..

[130] Göksen G., Fabra M.J., Pérez-Cataluña A., Ekiz H.I., Sanchez G., López-Rubio A. (2021). Biodegradable active food packaging structures based on hybrid cross-linked electrospun polyvinyl alcohol fibers containing essential oils and their application in the preservation of chicken breast fillets. Food Packag. Shelf Life.

[131] Hanani Z.N., Husna A.A., Syahida S.N., Khaizura M.N., Jamilah B. (2018). Effect of different fruit peels on the functional properties of gelatin/polyethylene bilayer films for active packaging. Food Packag. Shelf Life.

[132] Arkoun M., Daigle F., Holley R.A., Heuzey M.C., Ajji A. (2018). Chitosan-based nanofibers as bioactive meat packaging materials. Packag. Technol. Sci..

[133] Priya N.V., Vinitha U.G., Sundaram M.M. (2021). Preparation of chitosan-based antimicrobial active food packaging film incorporated with Plectranthus amboinicus essential oil. Biocatal. Agric. Biotechnol..

[134] Fiore A., Park S., Volpe S., Torrieri E., Masi P. (2021). Active packaging based on PLA and chitosan-caseinate enriched rosemary essential oil coating for fresh minced chicken breast application. Food Packag. Shelf Life.

[135] Go E.J., Song K.B. (2019). Antioxidant properties of rye starch films containing rosehip extract and their application in packaging of chicken breast. Starch Stärke.

[136] Hosseinzadeh S., Partovi R., Talebi F., Babaei A. (2020). Chitosan/TiO 2 nanoparticle/Cymbopogon citratus essential oil film as food packaging material: Physico-mechanical properties and its effects on microbial, chemical, and organoleptic quality of minced meat during refrigeration. J. Food Process. Preserv..

[137] Valdés A., Mellinas A.C., Ramos M., Burgos N., Jiménez A., Garrigós M.C. (2015). Use of herbs, spices and their bioactive compounds in active food packaging. RSC Adv..

[138] Gharsallaoui A., Joly C., Oulahal N., Degraeve P. (2013). Nisin as a Food Preservative: Part 2: Antimicrobial Polymer Materials Containing Nisin. Crit. Rev. Food Sci. Nutr..

[139] Li X., Xiao N., Xiao G., Bai W., Zhang X., Zhao W. (2021). Lemon essential oil/vermiculite encapsulated in electrospun konjac glucomannan-grafted-poly (acrylic acid)/polyvinyl alcohol bacteriostatic pad: Sustained control release and its application in food preservation. Food Chem..

[140] Hosseini F., Miri M.A., Najafi M., Soleimanifard S., Aran M. (2021). Encapsulation of rosemary essential oil in zein by electrospinning technique. J. Food Sci..

[141] Wen P., Zhu D.-H., Wu H., Zong M.-H., Jing Y.-R., Han S.-Y. (2016). Encapsulation of cinnamon essential oil in electrospun nanofibrous film for active food packaging. Food Control.

[142] Çallıoğlu F.C., Güler H.K., Çetin E.S., Çallıoğlu F.C.Z. (2019). Emulsion electrospinning of bicomponent poly (*Vinyl pyrrolidone*)/gelatin nanofibers with thyme essential oil. Mater. Res. Express.

[143] Can F.O., Durak M.Z. (2021). Encapsulation of Lemongrass Oil for Antimicrobial and Biodegradable Food Packaging Applications. Sci. Adv. Mater..

[144] Figueroa-Lopez K.J., Torres-Giner S., Enescu D., Cabedo L., Cerqueira M., Pastrana L.M., Lagaron J.M. (2020). Electrospun Active Biopapers of Food Waste Derived Poly(3-hydroxybutyrate-co-3-hydroxyvalerate) with Short-Term and Long-Term Antimicrobial Performance. Nanomaterials.

[145] Leena M.M., Yoha K.S., Moses J.A., Anandharamakrishnan C. (2021). Electrospun nanofibrous membrane for filtration of coconut neera. Nanotechnol. Environ. Eng..

[146] Aytac Z., Huang R., Vaze N., Xu T., Eitzer B.D., Krol W., Mac Queen L.A., Chang H., Bousfield D.W., Chan-Park M.B. (2020). Development of Biodegradable and Antimicrobial Electrospun Zein Fibers for Food Packaging. ACS Sustain. Chem. Eng..

[147] Alehosseini A., Gómez-Mascaraque L.G., Martínez-Sanz M., López-Rubio A. (2019). Electrospun curcumin-loaded protein nanofiber mats as active/bioactive coatings for food packaging applications. Food Hydrocoll..

[148] Ramalingam R., Dhand C., Leung C.M., Ong S.T., Annamalai S.K., Kamruddin M., Verma N.K., Ramakrishna S., Lakshminarayanan R., Arunachalam K.D. (2019). Antimicrobial properties and biocompatibility of electrospun poly-ε-caprolactone fibrous mats containing Gymnema sylvestre leaf extract. Mater. Sci. Eng. C.

[149] Hamdan N., Yamin A., Hamid S.A., Khodir W.K.W.A., Guarino V. (2021). Functionalized Antimicrobial Nanofibers: Design Criteria and Recent Advances. J. Funct. Biomater..

[150] Altan A., Aytac Z., Uyar T. (2018). Carvacrol loaded electrospun fibrous films from zein and poly(*Lactic acid*) for active food packaging. Food Hydrocoll..

[151] Padil V.V.T., Senan C., Wacławek S., Černík M., Agarwal S., Varma R.S. (2019). Bioplastic Fibers from Gum Arabic for Greener Food Wrapping Applications. ACS Sustain. Chem. Eng..

[152] Mahcene Z., Khelil A., Hasni S., Akman P.K., Bozkurt F., Birech K., Goudjil M.B., Tornuk F. (2020). Development and characterization of sodium alginate based active edible films incorporated with essential oils of some medicinal plants. Int. J. Biol. Macromol..

[153] Bruni G.P., Acunha T., De Oliveira J.P., Fonseca L.M., Da Silva F.T., Guimarães V.M., Zavareze E.D.R. (2020). Electrospun protein fibers loaded with yerba mate extract for bioactive release in food packaging. J. Sci. Food Agric..

[154] Chisenga S.M., Tolesa G.N., Workneh T.S. (2020). Biodegradable Food Packaging Materials and Prospects of the Fourth Industrial Revolution for Tomato Fruit and Product Handling. Int. J. Food Sci..

[155] Nakajima H., Dijkstra P., Loos K. (2017). The Recent Developments in Biobased Polymers toward General and Engineering Applications: Polymers that are Upgraded from Biodegradable Polymers, Analogous to Petroleum-Derived Polymers, and Newly Developed. Polymers.

[156] Kumar T.S.M., Kumar K.S., Rajini N., Siengchin S., Ayrilmis N., Rajulu A.V. (2019). A comprehensive review of electrospun nanofibers: Food and packaging perspective. Compos. Part B Eng..

[157] Moreno A.M., Orqueda E.M., Gomez-Mascaraque L.G., Isla I.M., Lopez-Rubio A. (2019). Crosslinked electrospun zein-based food packaging coatings containing bioactive chilto fruit extracts. Food Hydrocoll..

[158] Tsai Y.-H., Yang Y.-N., Ho Y.-C., Tsai M.-L., Mi F.-L. (2018). Drug release and antioxidant/antibacterial activities of silymarin-zein nanoparticle/bacterial cellulose nanofiber composite films. Carbohydr. Polym..

[159] Nilsen-Nygaard J., Fernández E.N., Radusin T., Rotabakk B.T., Sarfraz J., Sharmin N., Sivertsvik M., Sone I., Pettersen M.K. (2021). Current status of biobased and biodegradable food packaging materials: Impact on food quality and effect of innovative processing technologies. Compr. Rev. Food Sci. Food Saf..

[160] Aslam M., Kalyar M.A., Raza Z.A. (2018). Polyvinyl alcohol: A review of research status and use of polyvinyl alcohol based nanocomposites. Polym. Eng. Sci..

[161] Tian H., Yan J., Rajulu A.V., Xiang A., Luo X. (2017). Fabrication and properties of polyvinyl alcohol/starch blend films: Effect of composition and humidity. Int. J. Biol. Macromol..

[162] Ali A., Ahmed S. (2018). A review on chitosan and its nanocomposites in drug delivery. Int. J. Biol. Macromol..

[163] Gutiérrez C., Tomy J. (2017). Chitosan applications for the food industry. Chitosan: Derivatives. Composites and Applications.

[164] Priyadarshi R., Rhim J.-W. (2020). Chitosan-based biodegradable functional films for food packaging applications. Innov. Food Sci. Emerg. Technol..

[165] Ferreira D.C.M., de Souza A.L., da Silveira J.V.W., Marim B.M., Giraldo G.A.G., Mantovan J., Mali S., Pelissari F.M., Abd-Elsalam K.A. (2020). Chapter 17—Chitosan nanocomposites for food packaging applications. Multifunctional Hybrid Nanomaterials for Sustainable Agri-Food and Ecosystems.

[166] Ishkeh S., Shirzad H., Asghari M., Alirezalu A., Pateiro M., Lorenzo J. (2021). Effect of Chitosan Nanoemulsion on Enhancing the Phytochemical Contents, Health-Promoting Components, and Shelf Life of Raspberry (Rubus sanctus Schreber). Appl. Sci..

[167] Hao W., Li K., Ma Y., Li R., Xing R., Yu H., Li P. (2021). Preparation and Antioxidant Activity of Chitosan Dimers with Different Sequences. Mar. Drugs.

[168] Gumienna M., Górna B. (2021). Antimicrobial Food Packaging with Biodegradable Polymers and Bacteriocins. Molecules.

[169] Filho J.G.D.O., De Deus I.P.B., Valadares A.C.F., Fernandes C.C., Estevam E.B.B., Egea M.B. (2020). Chitosan Film with Citrus limonia Essential Oil: Physical and Morphological Properties and Antibacterial Activity. Colloids Interfaces.

[170] Liu F., Liu Y., Sun Z., Wang D., Wu H., Du L., Wang D. (2020). Preparation and antibacterial properties of ε-polylysine-containing gelatin/chitosan nanofiber films. Int. J. Biol. Macromol..

[171] De Farias B.S., Junior T.R.S.A.C., de Almeida Pinto L.A. (2019). Chitosan-functionalized nanofibers: A comprehensive review on challenges and prospects for food applications. Int. J. Biol. Macromol..

[172] Ardekani-Zadeh A.H., Hosseini S.F. (2019). Electrospun essential oil-doped chitosan/poly(ε-caprolactone) hybrid nanofibrous mats for antimicrobial food biopackaging exploits. Carbohydr. Polym..

[173] Suryani S., Rihayat T., Nurhanifa N., Riskina S. (2020). Modification of Poly Lactid Acid (PLA)/Chitosan with cinnamon essential oil for antibacterial applications. IOP Conference Series: Materials Science and Engineering.

[174] Ebrahimzadeh S., Bari M.R., Hamishehkar H., Kafil H.S., Lim L.T. (2021). Essential oils-loaded electrospun chitosan-poly (vinyl alcohol) nonwovens laminated Hamishehkar, on chitosan film as bilayer bioactive edible films. LWT.

[175] Sun Y., Cheng S., Lu W., Wang Y., Zhang P., Yao Q. (2019). Electrospun fibers and their application in drug controlled release, biological dressings, tissue repair, and enzyme immobilization. RSC Adv..

[176] Torkamani A.E., Syahariza Z.A., Norziah M.H., Wan A.K.M., Juliano P. (2018). Encapsulation of polyphenolic antioxidants obtained from Momordica charantia fruit within zein/gelatin shell core fibers via coaxial electrospinning. Food Biosci..

[177] Ghorani B., Tucker N. (2015). Fundamentals of electrospinning as a novel delivery vehicle for bioactive compounds in food nanotechnology. Food Hydrocoll..

[178] Tampau A., González-Martínez C., Chiralt A. (2018). Release kinetics and antimicrobial properties of carvacrol encapsulated in electrospun poly-(ε-caprolactone) nanofibres. Application in starch multilayer films. Food Hydrocoll..

[179] Yao Z., Chen S., Ahmad Z., Huang J., Chang M., Li J. (2017). Essential Oil Bioactive Fibrous Membranes Prepared via Coaxial Electrospinning. J. Food Sci..

[180] Tran P., Duan W., Lee B.-J., Tran T.T. (2019). The use of zein in the controlled release of poorly water-soluble drugs. Int. J. Pharm..

[181] Gómez-Mascaraque L.G., Sipoli C.C., de La Torre L.G., López-Rubio A. (2017). Microencapsulation structures based on protein-coated liposomes obtained through electrospraying for the stabilization and improved bioaccessibility of curcumin. Food Chem..

[182] Hadad S., Goli S.A.H. (2019). Improving Oxidative Stability of Flaxseed Oil by Encapsulation in Electrospun Flaxseed Mucilage Nanofiber. Food Bioprocess Technol..

[183] Vishwakarma G.S., Gautam N., Babu J.N., Mittal S., Jaitak V. (2016). Polymeric Encapsulates of Essential Oils and Their Constituents: A Review of Preparation Techniques, Characterization, and Sustainable Release Mechanisms. Polym. Rev..

[184] Pithanthanakul U., Vatanyoopaisarn S., Thumthanaruk B., Puttanlek C., Uttapap D., Kietthanakorn B., Rungsardthong V. (2021). Encapsulation of fragrances in zein nanoparticles and use as fabric softener for textile application. Flavour Fragr. J..

[185] Rather A., Wani T., Khan R., Pant B., Park M., Sheikh F. (2021). Prospects of Polymeric Nanofibers Loaded with Essential Oils for Biomedical and Food-Packaging Applications. Int. J. Mol. Sci..

[186] Pillay V., Dott C., Choonara Y., Tyagi C., Tomar L., Kumar P., du Toit L., Ndesendo V.M.K. (2013). A Review of the Effect of Processing Variables on the Fabrication of Electrospun Nanofibers for Drug Delivery Applications. J. Nanomater..

[187] Lavoine N., Guillard V., Desloges I., Gontard N., Bras J. (2016). Active bio-based food-packaging: Diffusion and release of active substances through and from cellulose nanofiber coating toward food-packaging design. Carbohydr. Polym..

[188] Wang P., Mele E. (2018). Effect of Antibacterial Plant Extracts on the Morphology of Electrospun Poly(*Lactic Acid*) Fibres. Materials.

[189] Milanesi G., Vigani B., Rossi S., Sandri G., Mele E. (2021). Chitosan-Coated Poly(*Lactic acid*) Nanofibres Loaded with Essential Oils for Wound Healing. Polymers.

[190] Melendez-Rodriguez B., Figueroa-Lopez K.J., Bernardos A., Martínez-Máñez R., Cabedo L., Torres-Giner S., Lagaron J.M. (2019). Electrospun Antimicrobial Films of Poly(3-hydroxybutyrate-co-3-hydroxyvalerate) Containing Eugenol Essential Oil Encapsulated in Mesoporous Silica Nanoparticles. Nanomaterials.

[191] Esfanjani A.F., Assadpour E., Jafari S.M. (2018). Improving the bioavailability of phenolic compounds by loading them within lipid-based nanocarriers. Trends Food Sci. Technol..

[192] Aytac Z., Ipek S., Durgun E., Uyar T. (2017). Antioxidant electrospun zein nanofibrous web encapsulating quercetin/cyclodextrin inclusion complex. J. Mater. Sci..

[193] Cui H., Bai M., Li C., Liu R., Lin L. (2018). Fabrication of chitosan nanofibers containing tea tree oil liposomes against Salmonella spp. in chicken. LWT.

[194] Liu Y., Wang S., Zhang R., Lan W., Qin W. (2017). Development of Poly(*Lactic acid*)/Chitosan Fibers Loaded with Essential Oil for Antimicrobial Applications. Nanomaterials.

[195] Babitha S., Rachita L., Karthikeyan K., Shoba E., Janani I., Poornima B., Sai K.P. (2017). Electrospun protein nanofibers in healthcare: A review. Int. J. Pharm..

[196] Deng L., Li Y., Feng F., Wu D., Zhang H. (2019). Encapsulation of allopurinol by glucose cross-linked gelatin/zein nanofibers: Characterization and release behavior. Food Hydrocoll..

[197] Grkovic M., Stojanovic D.B., Pavlovic V.B., Rajilic-Stojanovic M., Bjelovic M., Uskokovic P.S. (2017). Improvement of mechanical properties and antibacterial activity of crosslinked electrospun chitosan/poly (ethylene oxide) nanofibers. Compos. Part B Eng..

[198] Dodero A., Scarfi S., Mirata S., Sionkowska A., Vicini S., Alloisio M., Castellano M. (2021). Effect of Crosslinking Type on the Physical-Chemical Properties and Biocompatibility of Chitosan-Based Electrospun Membranes. Polymers.

[199] Ehrmann A. (2021). Non-Toxic Crosslinking of Electrospun Gelatin Nanofibers for Tissue Engineering and Biomedicine—A Review. Polymers.

[200] Chen X., Meng J., Xu H., Shinoda M., Kishimoto M., Sakurai S., Yamane H. (2021). Fabrication and Properties of Electrospun Collagen Tubular Scaffold Crosslinked by Physical and Chemical Treatments. Polymers.

[201] Santiago-Morales J., Amariei G., Letón P., Rosal R. (2016). Antimicrobial activity of poly(*Vinyl alcohol*)-poly(*Acrylic acid*) electrospun nanofibers. Colloids Surf. B Biointerfaces.

[202] Wardhani R.A.K., Asri L.A.T.W., Rachmawati H., Khairurrijal K., Purwasasmita B.S. (2020). Physical-Chemical Crosslinked Electrospun Colocasia esculenta Tuber Protein-Chitosan-Poly(*Ethylene Oxide*) Nanofibers with Antibacterial Activity and Cytocompatibility. Int. J. Nanomed..

[203] Nataraj D., Reddy R., Reddy N. (2020). Crosslinking electrospun poly (vinyl) alcohol fibers with citric acid to impart aqueous stability for medical applications. Eur. Polym. J..

[204] Suganthi S., Vignesh S., Sundar J.K., Raj V. (2020). Fabrication of PVA polymer films with improved antibacterial activity by fine-tuning via organic acids for food packaging applications. Appl. Water Sci..

[205] Çay A., Miraftab M. (2013). Properties of electrospun poly(*Vinyl alcohol*) hydrogel nanofibers crosslinked with 1,2,3,4-butanetetracarboxylic acid. J. Appl. Polym. Sci..

[206] Destaye A.G., Lin C.-K., Lee C.-K. (2013). Glutaraldehyde Vapor Cross-linked Nanofibrous PVA Mat with in Situ Formed Silver Nanoparticles. ACS Appl. Mater. Interfaces.

[207] Zhu L., Zaarour B., Jin X. (2019). Fabrication of perfect CMCS/PVA nanofibers for keeping food fresh via an in situ mixing electrospinning. Mater. Res. Express.

[208] Lamarra J., Calienni M.N., Rivero S., Pinotti A. (2020). Electrospun nanofibers of poly(*Vinyl alcohol*) and chitosan-based emulsions functionalized with cabreuva essential oil. Int. J. Biol. Macromol..

[209] Miraftab M., Saifullah A.N.M., Çay A. (2014). Physical stabilisation of electrospun poly(*Vinyl alcohol*) nanofibres: Comparative study on methanol and heat-based crosslinking. J. Mater. Sci..

[210] Lin L., Gu Y., Cui H. (2018). Novel electrospun gelatin-glycerin-ε-Poly-lysine nanofibers for controlling Listeria monocytogenes on beef. Food Packag. Shelf Life.

[211] Leung H.-W. (2001). Ecotoxicology of Glutaraldehyde: Review of Environmental Fate and Effects Studies. Ecotoxicol. Environ. Saf..

[212] Thakur A., Wanchoo R.K., Hardeep S.K.S. (2013). Chitosan Hydrogel Beads: A Comparative Study with Glutaraldehyde, Epichlorohydrin and Genipin as Crosslinkers. J. Polym. Mater..

[213] Arteche Pujana M., Pérez-Álvarez L., Cesteros Iturbe L.C., Katime I. (2013). Biodegradable chitosan nanogels crosslinked with genipin. Carbohydr. Polym..

[214] Chen J., Han S., Huang M., Li J., Zhou M., He J. (2020). Green crosslinked nanofibers membrane based on CS/PVA combined with polybasic organic acid for tympanic membrane repair. Int. J. Polym. Mater..

[215] Wu X., Dai H., Xu C., Liu L., Li S. (2019). Citric acid modification of a polymer exhibits antioxidant and anti-inflammatory properties in stem cells and tissues. J. Biomed. Mater. Res. Part A.

[216] Suganthi S., Mohanapriya S., Raj V., Kanaga S., Dhandapani R., Vignesh S., Sundar J.K. (2018). Tunable Physicochemical and Bactericidal Activity of Multicarboxylic-Acids-Crosslinked Polyvinyl Alcohol Membrane for Food Packaging Applications. ChemistrySelect.

[217] Yu D., Feng Y., Xu J., Kong B., Liu Q., Wang H. (2021). Fabrication, characterization, and antibacterial properties of citric acid crosslinked PVA electrospun microfibre mats for active food packaging. Packag. Technol. Sci..

[218] Zhu Y., Cui H., Li C., Lin L. (2019). A novel polyethylene oxide/Dendrobium officinale nanofiber: Preparation, characterization and application in pork packaging. Food Packag. Shelf Life.

[219] Imre B., García L., Puglia D., Vilaplana F. (2019). Reactive compatibilization of plant polysaccharides and biobased polymers: Review on current strategies, expectations and reality. Carbohydr. Polym..

[220] Anvari M., Chung D. (2016). Dynamic rheological and structural characterization of fish gelatin—Gum arabic coacervate gels cross-linked by tannic acid. Food Hydrocoll..

[221] Jakobek L. (2015). Interactions of polyphenols with carbohydrates, lipids and proteins. Food Chem..

[222] Vilas-Boas A., Pintado M., Oliveira A. (2021). Natural Bioactive Compounds from Food Waste: Toxicity and Safety Concerns. Foods.

[223] Torres F.G., Troncoso O.P., Pisani A., Gatto F., Bardi G. (2019). Natural Polysaccharide Nanomaterials: An Overview of Their Immunological Properties. Int. J. Mol. Sci..

[224] Wang Z., Sun Q., Zhang H., Wang J., Fu Q., Qiao H., Wang Q. (2021). Insight into antibacterial mechanism of polysaccharides: A review. LWT.

[225] Xia G.-X., Wu Y.-M., Bi Y.-F., Chen K., Zhang W.-W., Liu S.-Q., Zhang W.-J., Liu R.-H. (2021). Antimicrobial Properties and Application of Polysaccharides and Their Derivatives. Chin. J. Polym. Sci..

[226] Sakib M.N., Mallik A.K., Rahman M.M. (2021). Update on chitosan-based electrospun nanofibers for wastewater treatment: A review. Carbohydr. Polym. Technol. Appl..

[227] Tien N., Lyngstadaas S., Mano J., Blaker J., Haugen H. (2021). Recent Developments in Chitosan-Based Micro/Nanofibers for Sustainable Food Packaging, Smart Textiles, Cosmeceuticals, and Biomedical Applications. Molecules.

[228] Liu Y., Wang D., Sun Z., Liu F., Du L., Wang D. (2021). Preparation and characterization of gelatin/chitosan/3-phenylacetic acid food-packaging nanofiber antibacterial films by electrospinning. Int. J. Biol. Macromol..

[229] Deng L., Taxipalati M., Zhang A., Que F., Wei H., Feng F., Zhang H. (2018). Electrospun Chitosan/Poly(*Ethylene oxide*)/Lauric Arginate Nanofibrous Film with Enhanced Antimicrobial Activity. J. Agric. Food Chem..

[230] Pandey V.K., Upadhyay S.N., Niranjan K., Mishra P.K. (2020). Antimicrobial biodegradable chitosan-based composite Nano-layers for food packaging. Int. J. Biol. Macromol..

[231] Dehghani S., Rezaei K., Hamishehkar H., Oromiehie A. (2021). The effect of electrospun polylactic acid/chitosan nanofibers on the low density polyethylene/ploy lactic acid film as bilayer antibacterial active packaging films. J. Food Process. Preserv..

[232] Agarwal A., Raheja A., Natarajan T., Chandra T. (2014). Effect of electrospun montmorillonite-nylon 6 nanofibrous membrane coated packaging on potato chips and bread. Innov. Food Sci. Emerg. Technol..

[233] Lopresti F., Pavia F.C., Ceraulo M., Capuana E., Brucato V., Ghersi G., Botta L., La Carrubba V. (2021). Physical and biological properties of electrospun poly(d, l-lactide)/nanoclay and poly(d, l-lactide)/nanosilica nanofibrous scaffold for bone tissue engineering. J. Biomed. Mater. Res. Part A.

[234] Othman S.H., Ling H.N., Talib R.A., Naim M.N., Risyon N.P. (2019). Saifullah PLA/MMT and PLA/Halloysite Bio-Nanocomposite Films: Mechanical, Barrier, and Transparency. J. Nano Res..

[235] Wahab A., Luming L., Matin A., Karim M.R., Aijaz M.O., Alharbi H.F., Abdala A., Haque R. (2021). Silver Micro-Nanoparticle-Based Nanoarchitectures: Synthesis Routes, Biomedical Applications, and Mechanisms of Action. Polymers.

[236] Ferdous Z., Nemmar A. (2020). Health Impact of Silver Nanoparticles: A Review of the Biodistribution and Toxicity Following Various Routes of Exposure. Int. J. Mol. Sci..

[237] Kowsalya E., Mosa Christas K., Balashanmugam P., Rani J.C. (2019). Biocompatible silver nanoparticles.poly (*Vinyl alcohol*) electrospun nanofibers for potential antimicrobial food packaging applications. Food Packag. Shelf Life.

[238] Tarus B.K., Mwasiagi J.I., Fadel N., Al-Oufy A., El Messiry M. (2019). Electrospun cellulose acetate and poly(*Vinyl chloride*) nanofiber mats containing silver nanoparticles for antifungi packaging. SN Appl. Sci..

[239] Segala K., Nista S.V.G., Cordi L., Bizarria M.T.M., de Ávila Júnior J., Kleinubing S.A., Cruz D.C., Brocchi M., Lona L.M.F., Caballero N.E.D. (2015). Silver nanoparticles incorporated into nanostructured biopolymer membranes produced by electrospinning: A study of antimicrobial activity. Braz. J. Pharm. Sci..

[240] Zhan F., Yan X., Sheng F., Li B. (2020). Facile in situ synthesis of silver nanoparticles on tannic acid/zein electrospun membranes and their antibacterial, catalytic and antioxidant activities. Food Chem..

[241] Munteanu B.S., Aytac Z., Pricope G.M., Uyar T., Vasile C. (2014). Polylactic acid (PLA)/Silver-NP/VitaminE bionanocomposite electrospun nanofibers with antibacterial and antioxidant activity. J. Nanoparticle Res..

[242] Zhang R., Lan W., Ji T., Sameen D.E., Ahmed S., Qin W., Liu Y. (2021). Development of polylactic acid. ZnO composite membranes prepared by ultrasonication and electrospinning for food packaging. LWT.

[243] Estevez-Areco S., Guz L., Candal R., Goyanes S. (2020). Active bilayer films based on cassava starch incorporating ZnO nanorods and PVA electrospun mats containing rosemary extract. Food Hydrocoll..

[244] Pereira L.A.S., Bemfeito R.M., Bemfeito C.M., Silva P.D.C.E., Rodrigues J.F., Gonçalves M.C., Pinheiro A.C.M., Piccoli R.H. (2020). Acceptability of low-sodium mozzarella coated with zein and essential oils. Br. Food J..

[245] Kirkin C., Inbat S.M., Nikolov D., Yildirim S. (2019). Effects of tarragon essential oil on some characteristics of frankfurter type sausages. AIMS Agric. Food.

[246] Ajayi-Moses O.B., Ogidi C.O., Akinyele B.J. (2019). Bioactivity of Citrus essential oils (CEOs) against microorganisms associated with spoilage of some fruits. Chem. Biol. Technol. Agric..

[247] Serwańska-Leja K., Drożdżyńska A., Majcher M., Kowalczewski P.Ł., Czaczyk K. (2019). Influence of sub-inhibitory concentration of selected plant essential oils on the physical and biochemical properties of Pseudomonas orientalis. Open Chem..

[248] Laranjo M., Fernández-León A.M., Agulheiro-Santos A.C., Potes M.E., Elias M. (2019). Essential oils of aromatic and medicinal plants play a role in food safety. J. Food Process. Preserv..

[249] Sedki A.G., El-Zainy A.R., Rajab B.T. (2020). Thyme and Clove Essential Oils as Antioxidants and Antimicrobial in Beef Sausage. J. Food Nutr. Sci..

[250] Stoleru E., Vasile C., Irimia A., Brebu M. (2021). Towards a Bioactive Food Packaging: Poly(*Lactic Acid*) Surface Functionalized by Chitosan Coating Embedding Clove and Argan Oils. Molecules.

[251] Bahurmiz O.M., Ahmad R., Ismail N., Adzitey F., Sulaiman S.-F. (2020). Antimicrobial Activity of Selected Essential Oils on Pseudomonas Species Associated with Spoilage of Fish with Emphasis on Cinnamon Essential Oil. J. Aquat. Food Prod. Technol..

[252] Fattahi R., Ghanbarzadeh B., Dehghannya J., Hosseini M., Falcone P.M. (2020). The effect of Macro and Nano-emulsions of cinnamon essential oil on the properties of edible active films. Food Sci. Nutr..

[253] György É., Laslo É., Kuzman I.H., Dezső András C. (2020). The effect of essential oils and their combinations on bacteria from the surface of fresh vegetables. Food Sci. Nutr..

[254] Bazana M.T., Codevilla C.F., de Menezes C.R. (2019). Nanoencapsulation of bioactive compounds: Challenges and perspectives. Curr. Opin. Food Sci..

